# Neglecting students’ socio-emotional skills magnified learning losses during the pandemic

**DOI:** 10.1038/s41539-024-00235-9

**Published:** 2024-04-09

**Authors:** Guilherme Lichand, Julien Christen, Eppie Van Egeraat

**Affiliations:** 1 Graduate School of Education, Stanford University, 520 Galvez Mall, CERAS 519, Stanford, CA 94305 USA; 2 Laterite, Kampala, Uganda; 3 Department of Economics, NHH, Bergen, Norway

**Keywords:** Education, Economics

## Abstract

Did the dramatic learning losses from remote learning in the context of COVID-19 stem at least partly from schools having overlooked students’ socio-emotional skills—such as their ability to self-regulate emotions, their mental models, motivation, and grit—during the emergency transition to remote learning? We study this question using a cluster-randomized control trial with 18,256 high-school students across 87 schools in the State of Goiás, Brazil. The intervention sent behavioral nudges through text messages to students or their caregivers, targeting their socio-emotional skills during remote learning. Here we show that these messages significantly increased standardized test scores relative to the control group, preventing 7.5% of learning losses in math and 24% in Portuguese, consistent with the hypothesis that neglecting students’ socio-emotional skills magnified learning losses during the pandemic.

## Introduction

The impacts of remote learning on educational outcomes in the context of COVID-19 have been shown to be nothing short of catastrophic where schools remained closed for long^1^. In low- and middle-income countries, at the same time as in-person classes were suspended for longer than anywhere else, the conditions to study remotely were the most precarious, and the health and economic impacts of the pandemic, the most brutal^2^. Even though some locations and age ranges suffered less pronounced learning losses on average, particularly within Europe and the US, in almost every case poorer students were still significantly impacted, widening pre-pandemic educational inequalities^3^. For Brazil, in particular, remote learning during the pandemic has been causally associated with dramatic learning losses and a sharp increase in student dropout risk^4^. According to data from Goiás State, the setting of our study, high-school senior students’ average proficiency backtracked 2.84 years in math and 2.25 years in Portuguese in the absence of in-person classes.

Beyond learning outcomes, a growing literature documents that students’ mental health and socio-emotional skills were also hurt throughout the pandemic (e.g., ref. ^5^). Such impacts matter in and of themselves, but also because of their intricate connections to learning in and out of school. The ability to regulate emotions and navigate complex contextual changes are at the core of students’ learning experiences, particularly during adolescence. Neurobiological changes during puberty redirect adolescents’ attention and motivational salience, with status-seeking behaviors, romantic interests and peer pressure often getting in the way of attending classes^6^. As a result, adolescents are the ones most likely to disengage from studies, and to ultimately abandon school^7^. Consistent with these neurobiological mechanisms, socio-emotional skills had been previously shown to be key predictors of learning during in-person classes: grit (e.g., refs. ^8,9^), self-regulation (e.g., ref. ^10^), motivation (e.g., ref. ^11^), and a growth mindset (e.g., ref. ^12^), all have been linked to better educational outcomes, both in observational and experimental studies. As such, it is natural to hypothesize, on the one hand, that the worsening of students’ socio-emotional skills might have magnified learning losses during remote learning; in effect, motivation, grit, self-regulation and a growth mindset might be even more important without the structure provided by in-person classes and without adequate conditions to focus on academic activities. On the other hand, these skills might actually have played a less prominent role during remote learning, in the absence of face-to-face interactions, social pressure and other sources of motivational salience that often detract from learning, especially during adolescence^6^.

Which force dominated during the pandemic? Providing a causal answer to this question is challenging. The evidence that both socio-emotional skills and learning outcomes deteriorated during remote learning is not sufficient (and not even necessary) to establish causality. The latter requires exogenous variation in students’ socio-emotional skills during the pandemic.

Providing a causal answer this question is also important. Despite concerted efforts to adapt the curriculum to the new circumstances and instruction to new media, students’ socio-emotional skills were largely overlooked during the emergency transition to remote learning. Worse still, even in the aftermath of the pandemic, it remains the case that remedial policies prioritized by low- and middle-income countries are largely focused on curricular content; in Brazil, a recent nationally representative survey found that 60% of students were enrolled in schools that did not offer resources for psychological support^13^.

The contribution of this paper is to leverage a large-scale experiment to study this question. Using a cluster-randomized control trial in the State of Goiás, Brazil, we investigate whether behavioral nudges to high-school students throughout remote learning mitigated learning losses during the pandemic. These nudges, sent through text messages to students or their parents over the course of nearly 12 months (over the 2020 and 2021 academic years), targeted students’ socio-emotional skills; in particular, messages tried to motivate students to stay engaged with school activities during remote learning, to support them in regulating negative emotions, to foster a growth mindset, and to develop grit. We estimate treatment effects on math and Portuguese standardized test scores at the end of the intervention. Extrapolating from previous trends in school-level standardized test scores, we are able to quantify the extent to which neglecting students socio-emotional skills magnified learning losses during remote learning. We also estimate heterogeneous treatment effects by student and school characteristics.

Importantly, we document that the impacts of the intervention were not merely the results of experimenter demand effects—e.g., in case text messages merely increased the salience of school activities, led students to infer social expectations about school effort, or led teachers to exert higher effort in schools targeted by the intervention—, thanks to the fact that, within the treatment group, the specific script of the intervention was randomly assigned *at the student level* (unbeknownst to teachers). Taking advantage of that additional experiment, we show that the content of the text messages ultimately matters.

## Results

### Treatment effects on school-level proficiency

Using data on the 2018, 2019, and 2021 State-wide proficiency exams, Table 1 estimates treatment effects on proficiency levels and on an indicator variable of whether schools are rated as below basic proficiency levels (=1 if sub-proficient, and 0 otherwise).

**Table 1:** Treatment effects on school-level test scores and share of sub-proficient schools at State-wide exam

	Test scores					Share of sub-proficient	
	Grade 12				Grade 9		
	All	Portuguese	Math	All	All	All	All
	(1)	(2)	(3)	(4)	(5)	(6)	(7)
Nudges	2.345	3.455	1.236	3.003		−0.087*	−0.116*
	(3.244)	(3.479)	(3.592)	(3.678)		(0.048)	(0.066)
2019	3.038	3.168	2.908	3.781	6.918***	−0.054*	−0.076**
	(1.973)	(2.033)	(2.254)	(2.580)	(2.355)	(0.028)	(0.038)
2021	−9.314***	−7.914***	−10.714***	−8.931***	−0.897	0.055	0.040
	(2.857)	(2.995)	(3.131)	(3-167)	(3407)	(0.043)	(0.047)
Placebo period				−1.352			0.058
				(3–881)			(0.060)
Placebo grade					−1.234		0.019
					(4.001)		(0.101)
School fixed effects	✓	✓	✓	✓	✓	✓	✓
Subject fixed effects	✓			✓	✓	✓	✓
Grade fixed effects							✓
Observations	340	170	170	340	188	528	528
Control mean (2018)	282.509	281.073	283.945	282.509	252.414	0.171	0.171

Ordinary Least Squares regressions of school-level average standardized test scores (for grades 5, 9 and 12) on an indicator of whether the school was part of the treatment group of the intervention in that year (Nudges = 1 in 2021, and 0 otherwise). Columns 1 through 4 restrict attention to grade 12 while Column 5 turns to grade 9. Columns 2 and 3 focus on Portuguese and math, respectively, while Columns 1, 4 and 5 consider overall test scores. Columns 6 and 7 consider treatment effects on an indicator of whether school-level average standardized test scores were below the minimum proficiency level set by the State (=1 if test scores <250). Placebo period = 1 if Nudges = 1, grade = 12 and year >2019. Placebo grade = 1 if Nudges = 1, grade = 9 and year = 2021. Standard errors clustered at the school level in parentheses. *p* < 0.01, **p < 0.05, *p < 0.1.

**Fig. 1:**
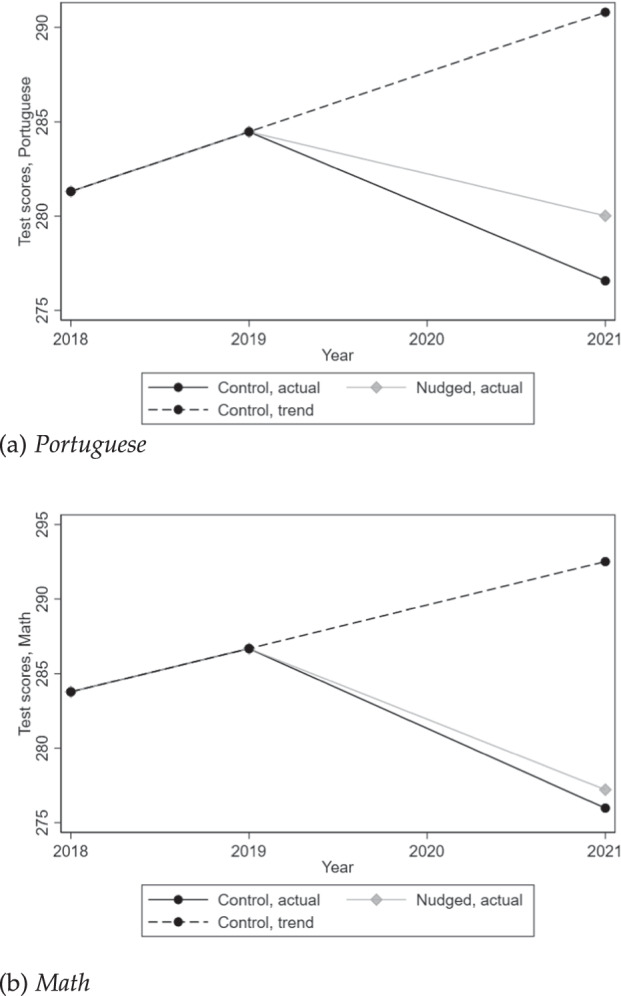
Average test scores in State-wide exams, separately for nudged and control schools. Yearly average testscores in Portuguese (**a**) and math (**b**) standardized exams between 2018 and 2021, for the treatment group (in light grey) and the control group (in black).

According to the table, learning deteriorated tremendously during the pandemic. Taking the 2018–2019 trend in proficiency levels as a counterfactual, high-school senior students’ average proficiency backtracked 2.84 years in math and 2.25 years in Portuguese. Nudges were able to prevent 24.2% of learning losses in Portuguese (column 2) and 7.5% of those in math (column 3), but these effect sizes are only imprecisely estimated: 90% CI (measured in raw test scores): [−2.3, 9.2] and [−4.7, 7.1], respectively, relative to average test scores of 281.1 for Portuguese and 283.9 for math. These trends and effect sizes are illustrated in Fig. 1. Back to the table, columns 4 and 5 rule out that the effects we estimate are merely driven by imbalances or experimenter demand effects (at least at the school level): the former documents that treated schools were *not* already improving relative to control schools before the onset of the intervention, and the latter, that 9th graders in treated schools did *not* improve relative to those in control schools in 2021.

**Fig. 2:**
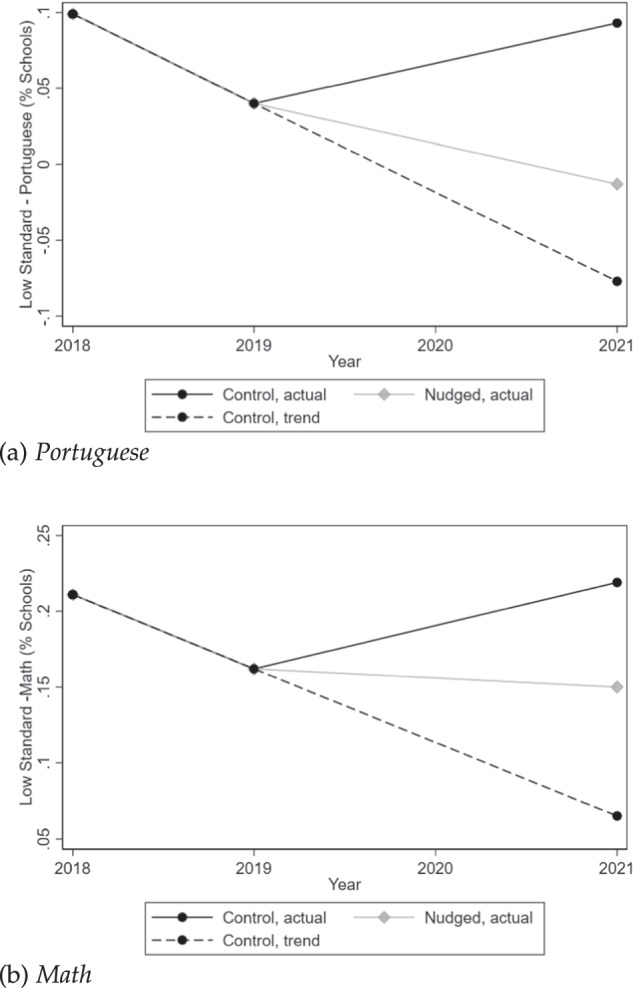
Share of schools below minimum proficiency levels in State-wide exams, separately for nudged and control schools. Yearly % of schools with average test scores below 250 in Portuguese (**a**) and math (**b**) standardized exams between 2018 and 2021, for the treatment group (in light grey) and the control group (in black).

The last two columns of the table turn to treatment effects on the share of sub-proficient schools across Portuguese and math. Nudges significantly decreased the share of schools with sub-proficient average test scores, from 17.1% to 8.4% (90% CI: [−0.166, −0.008]; column 7). Column 8 documents that treated schools were *not* improving relative to the control group before the onset of nudges, or in grades not targeted by the intervention. Trends and effect sizes for the share of sub-proficient schools are illustrated in Fig. 2 separately for Portuguese and math. Before the pandemic, State schools were on a rising trend: between 2018 and 2019, the share of sub-proficient schools had fallen from 21% to 16% in math, and from 10% to 4% in Portuguese—en route to eliminate sub-proficient standards in the following year. The pandemic dramatically reversed this trend: by 2021, that share was back up to 22% in math and 10% in Portuguese, in control schools. In treated schools, however, students continued to improve despite dire conditions: in that group, the share of sub-proficient schools reached less than 15% in math and 0% in Portuguese by 2021.

While the differences above are statistically significant, school-level averages are too aggregate to allow us to detect significant treatment effects on test scores. Moreover, aggregate results could mask selection effects in the case that nudges changed the composition of students across treated and control schools by affecting dropouts. For this reason, we next turn to student-level data from the additional standardized test.

### Treatment effects on student-level standardized test scores

#### Main effects

Table 2 estimates differences in standardized test scores across treatment and control students. Column 1 shows that nudges increased Portuguese standardized test scores by 0.21 s.d. (90% CI: [0.06, 0.35]). To benchmark that effect size, students with below-median Q1 Portuguese report card grades scored 0.31 s.d. below above-median ones in the control group. In other words, the effect size of nudges would be enough to close roughly 2/3 of the gap in test scores associated with differences in baseline achievement. When it comes to math, nudges also increased test scores, but the effect size was smaller and only imprecisely estimated (0.10 s.d. in column 2; 90% CI: [−0.05, 0.25]). The magnitude of this impact is, however, large; in fact, it is larger than effect sizes in most studies about the effects of educational nudges (~0.8–0.9 s.d.;^14^). Turning to the summary measure to account for multiple comparisons, the effect of nudges is still very large and significantly estimated in the pre-registered specification (0.19 s.d. in column 3; 90% CI: [0.03, 0.35]). Last, column 4 shows that results are robust to adjusting for any differences in baseline characteristics, and not an artifact of sample selection in combination with heterogeneity of treatment effects. This specification has only 960 (rather than 1,336) observations because 2021 State-wide proficiency levels are available for only 58 out of the 87 schools in our experiment. Controlling for student and school baseline characteristics and re-weighting observations by the inverse of their predicted probability of taking the standardized test (to ensure that our results are representative of the universe of students in full-time high schools in the State), we estimate that the intervention increased average standardized test scores by 0.195 s.d. (90% CI: [0.01, 0.38]).

**Table 2:** Treatment effects on standardized test scores

	Portuguese (std.)	Math (std.)	Test Score Summary Measure	
	(1)	(2)	(3)	(4)
Nudges	0.209**	0.098	0.189*	0.195*
	(0.088)	(0.093)	(0.098)	(0.111)
Observations	1337	1336	1336	960
R-squared	0.011	0.002	0.009	0.246
Controls	No	No	No	Yes
IPW	No	No	No	Yes

ITT estimates through Ordinary Least Squares regressions. In column 1, the dependent variable is the standardized test score in Portuguese; in column 2, that in math; and in columns 3 and 4, a summary measure of standardized test scores in Portuguese and math (following ref. ^38^). We standardize outcomes relative to the control group mean within each grade (such that the control group mean is 0 with standard deviation = 1). Nudges = 1 in schools where students were part of the treatment group of the intervention, and 0 otherwise. Controls include student gender, grade, phone ownership, a summary measure of Q1/2020 Portuguese and math report card grades, indicators for school-level presence of online activities, devices with internet access for students, internet access for student use, access to drinking water, adequate waste collection, bathrooms, libraries, science labs, computer labs and sports court (according to the 2019 school census), and the school-level Portuguese and math average test scores in the 2018 and 2019 SAEGO. Standard errors clustered at the school level in parentheses. Student-level probability of taking the Q2/2021 standardized test, used for computing inverse probability weights (IPW), predicted through a logit regression using the same covariates. Standard errors clustered at the school level in parentheses.

***p < 0.01, **p < 0.05, *p < 0.10.

#### Heterogeneous treatment effects

Next, we consider heterogeneity in the impacts of the intervention, estimating conditional average treatment effects (CATE) on the summary measure of standardized test scores by student and school characteristics. Table 3 estimates heterogeneous treatment effects by whether the student was enrolled in a school above the median of the 2019 State-wide exam proficiency level (based on a summary measure of Portuguese and math scores for high-school seniors; column 1); by whether the student was above the median baseline report card grades (mean of Q1/2020 math and Portuguese grades; column 2); by whether the student was a girl (column 3); by whether the student was enrolled in a school that offered online academic activities prior to the pandemic (according to the 2019 school census; column 4); and by whether the phone number targeted by the intervention belonged to the student him/herself (column 5). Across all columns, we control for all baseline covariates and re-weight observations by the inverse of their predicted probability of taking the standardized test.

**Table 3:** Heterogeneous treatment effects on standardized test scores

	Above-median school (52%)	Above-median student (57%)	Female (54%)	Previous online activities (81%)	Student owns phone (40%)
	(1)	(2)	(3)	(4)	(5)
Nudges	0.111	0.144	0.182	−0.108	0.077
	(0.144)	(0.132)	(0.132)	(0.229)	(0.123)
Characteristic	−0.246	−0.194	0.011	−0.036	−0.019
	(0.182)	(0.162)	(0.113)	(0.145)	(0.100)
Nudges x Characteristic	0.165	0.090	0.025	0.411	0.234*
	(0.175)	(0.145)	(0.153)	(0.263)	(0.138)
Observations	960	960	960	960	960
R-squared	0.249	0.248	0.246	0.252	0.250
Controls	Yes	Yes	Yes	Yes	Yes
IPW	Yes	Yes	Yes	Yes	Yes

ITT estimates through Ordinary Least Squares regressions. Across all columns, the dependent variable is a summary measure of standardized test scores in Portuguese and math (following ref. ^38^). We standardize the outcome relative to the control group mean within each grade (such that the control group mean is 0 with standard deviation = 1). Nudges = 1 in schools where students were part of the treatment group of the intervention, and 0 otherwise. Controls include student gender, grade, phone ownership, a summary measure of Q1/2020 Portuguese and math report card grades, indicators for school-level presence of online activities, devices with internet access for students, internet access for student use, access to drinking water, adequate waste collection, bathrooms, libraries, science labs, computer labs and sports court (according to the 2019 school census), and the school-level Portuguese and math average test scores in the 2018 and 2019 SAEGO. Student-level probability of taking the Q2/2021 standardized test, used for computing inverse probability weights (IPW), predicted through a logit regression using the same covariates. Each column estimates heterogeneity in treatment effects by a school or student characteristic: column 1, by whether the student was enrolled in a school above the median of the 2019 State-wide exam proficiency level (based on a summary measure of Portuguese and math scores for high-school seniors); column 2, by whether the student was above the median baseline report card grades (mean of Q1/2020 math and Portuguese grades); column 3, by whether the student was a girl; column 4, by whether the student was enrolled in a school that offered online academic activities prior to the pandemic (according to the 2019 school census); and column 5, by whether the phone number targeted by the intervention belonged to the student him/herself. Each column label displays the proportion of observations with that characteristic. Standard errors clustered at the school level in parentheses.

***p < 0.01, **p < 0.05, *p < 0.10.

Columns 1 and 2 showcase that schools and students who were relatively better off at baseline were more hard-hit by remote learning: test scores deteriorated more among these groups, to a large extent (by 0.25 s.d. more in above-median schools, and by 0.19 s.d. more among above-median students). As such, remote learning alleviated inequalities among high-school students in the State, but for the wrong reasons—a dramatic race to the bottom. While this is in contrast to evidence about the heterogeneous impacts of remote learning from high-income countries^1^ and even from different Brazilian States^4^, it matches the pattern observed in some low-income countries where only few students were able to thrive before the pandemic (e.g.,^15^). Although heterogeneous treatment effects are only imprecisely estimated, effect sizes are a lot larger precisely for the groups who lost the most ground in control schools: in each case, the intervention reduced differential losses by about half. The fact that treatment effects are larger for previously high-performing students is indicative of complementarities between curricular content and the socio-emotional skills targeted by the intervention.

When it comes to gender, column 3 documents that boys and girls did not systematically endure differential losses in control schools. Similarly, the effect of the intervention did not significantly vary by student gender.

Next, column 4 documents that, if anything, nudges’ effect size was actually negative for schools that did not offer online academic activities prior to the pandemic (although not statistically significant), while it was positive and much larger for those that did (−0.11 s.d. vs. 0.30 s.d., respectively). While we do not have enough statistical power to capture this difference with precision (also because only 20% of schools did not offer such activities in 2019), it is once again indicative of the complementary nature of curricular content and socio-emotional skills.

Last, even though targeting was not randomly assigned, we compare students for whom the Education Secretariat had access to their phone numbers (or only to their caregivers’ phone numbers) across the treatment and control groups; importantly, students’ phone ownership is balanced across treatment and control. Column 5 documents that targeting students mattered for the effects of nudges on standardized test scores: its effect size was significantly larger when students were targeted directly (0.31 s.d. vs. 0.08 s.d., a difference significant at the 10% level).

### Mechanisms

Did learning really improve as a result of the effects of the intervention on students’ socio-emotional skills? This section tackles that question from two perspectives. First, by documenting that nudges in fact had an impact on student motivation to stay engaged with school activities during remote learning. Second, by taking advantage of the content experiments to rule out alternative interpretations, such as experimenter demand effects or teachers’ strategic responses to the intervention.

#### Treatment effects on student motivation

Measuring students’ socio-emotional skills during the pandemic was challenging. Validated instruments to capture students’ self-regulation, grit or growth mindset are typically paper- or computer-based, rendering them unfeasible in a context of social distancing and limited connectivity such as the setting of our study.

Nevertheless, we combine administrative data on student participation in school activities (which was still being tracked before the winter break, immediately after the onset of the intervention) and SMS survey data to shed light on whether student motivation increased as a result of the intervention.

Supplementary Materials documents treatment effects on student participation in school activities and on their motivation to return to school by the time in-person classes would resume over the first 5 weeks after the onset of the intervention. While 7.21% of students in the control group had not followed remote learning activities right before the 2020 winter break, that figure was only 0.33% in the treatment group - an over 95% reduction (*p*-value < 0.001). Moreover, while lack of motivation to return to in-person classes was increasing quickly among control students—from 15% by the 2nd week of June to 39% by the 3rd week of July—, the intervention decreased that share by over 30% already by week 2. Effect sizes persisted into the winter break.

#### Treatment effects of content variations

Next, we draw on the content experiments, leveraging the student-level random assignment to different messages to shed light on whether the content of the intervention matters or, alternatively, whether the treatment effects that we document could be at least partly consistent with other interpretations, unrelated to students’ socio-emotional skills. If not all content works, and if some content variations are more effective than others, then we would falsify the hypothesis that treatment effects are merely reactions to higher salience of school activities or social expectations triggered by the intervention. Moreover, we would also falsify the hypothesis that they reflect teachers’ strategic responses to the intervention—as the latter were unaware of the student-level assignment in the context of the content experiments.

Table 4 estimates the treatment effects of being randomly assigned to messages alluding to social pressure, and of being randomly assigned to having messages about high school graduation framed either in terms of gains or losses. We allow for interactions between the effects of the two content experiments (since assignments were cross-randomized). In Table 4, even columns control for student and school baseline characteristics, and re-weight observations by the inverse of their predicted probability of taking the standardized test. In this table, we do not include school-level average baseline proficiency levels in the State-wide exam as a control in order not to unnecessarily decrease sample size (keeping 1304 observations in column 2, rather than only 960).

**Table 4:** Treatment effects of content variations on standardized test scores

	Test Score		Portuguese		Math	
	Summary	Measure	(std.)		(std.)	
	(1)	(2)	(3)	(4)	(5)	(6)
Framing Losses	0.175*	0.134	0.231**	0.218**	0.037	−0.029
	(0.089)	(0.081)	(0.095)	(0.090)	(0.101)	(0.105)
No Social Pressure	0.238**	0.219**	0.227**	0.234**	0.170*	0.121
	(0.100)	(0.094)	(0.111)	(0.116)	(0.100)	(0.089)
No Social Pressure x Framing Losses	−0.253*	−0.198*	−0.346**	−0.318**	−0.037	0.035
	(0.133)	(0.115)	(0.150)	(0.141)	(0.124)	(0.119)
Observations	1336	1304	1337	1305	1336	1304
R-squared	0.216	0.454	0.203	0.462	0.177	0.357
Classroom fixed-effects	Yes	Yes	Yes	Yes	Yes	Yes
Controls	No	Yes	No	Yes	No	Yes
IPW	No	Yes	No	Yes	No	Yes

ITT estimates through Ordinary Least Squares regressions. In columns 1 and 2, the dependent variable is a summary measure of standardized test scores in Portuguese and math (following ref. ^38^); in columns 3 and 4, the standardized test score in Portuguese; in columns 5 and 6, that in math. We standardize outcomes relative to the control group mean within each grade (such that the control group mean is 0 with standard deviation = 1). Nudges = 1 in schools where students were part of the treatment group of the intervention, and 0 otherwise. No Social Pressure = 1 for treated students who received messages stating the importance of returning to in-person classes without reference to their peers’ motivation to do so, and 0 otherwise (see Supplementary Table 2). Framing Loss = 1 for treated students who received messages framing the motivation to stay in school in terms of avoiding the loss of their high-school degree (see Supplemetary Table 2). Controls include student gender, grade, phone ownership, a summary measure of Q1/2020 Portuguese and math report card grades, indicators for school-level presence of online activities, devices with internet access for students, internet access for student use, access to drinking water, adequate waste collection, bathrooms, libraries, science labs, computer labs and sports court (according to the 2019 school census), and the school-level Portuguese and math average test scores in the 2018 and 2019 SAEGO. Student-level probability of taking the Q2/2021 standardized test, used for computing inverse probability weights (IPW), predicted through a logit regression using the same covariates. Standard errors (without clustering) in parentheses.

***p < 0.01, **p < 0.05, *p < 0.10.

Since the implementing partner only decided the content of the additional treatment arms after the roll-out of the experiment (after we had filed our pre-analysis plan), throughout these analyses we focus on whether effects *differ* across treatment arms—rather than on comparisons of each treatment arm with the control group (as these comparisons were not pre-registered). To do that, all columns absorb classroom fixed-effects, ensuring that all comparisons are within treated students.

The table shows that content matters for treatment effects. Framing the importance of staying in school in terms of avoiding losses increased standardized test scores by significantly more, driven by Portuguese test scores. Similarly, not resorting to social pressure in trying to keep students motivated during remote learning increased test scores by a much larger magnitude across both Portuguese and math, but mainly so for the latter. Interestingly, that was only the case for students who had not been exposed to the loss framing content variation. All in all, the fact that learning systematically responded to the content of the messages helps rule out alternative interpretations for our findings.

## Discussion

We find that behavioral nudges targeting students’ socio-emotional skills during the pandemic significantly decreased the share of schools below minimum proficiency levels relative to the control group from 17.1% to 8.4% (90% CI: [−0.166,−0.008]). At the school level, nudges prevented 24.2% of learning losses in Portuguese and 7.5% in math (although these effect sizes are only imprecisely estimated). Using student-level standardized test scores to increase precision, nudges increased Portuguese scores by 0.21 s.d. (90% CI: [0.06, 0.35])—enough to close 2/3 of the gap in scores associated to baseline differences in achievement—; in math, the effect size was smaller and only imprecisely estimated (0.10 s.d.; 90% CI: [−0.05, 0.25]). Student-level data also allow us to rule out that effects are driven by composition changes due to student dropouts during the pandemic—as the intervention did not systematically affect grade repetition or dropout risk, relative to the control group^16^.

While significant attention was devoted to pedagogical interventions from the outset of the emergency transition to remote learning, with a focus on mitigating learning deficits by the time children come back to in-person classes^17–19^, in reality, learning losses were still very large^4^, and a large fraction of children and adolescents might ultimately never return to school^2,20^. In contrast, support interventions targeting students’ socio-emotional skills, e.g., to motivate them to remain engaged with educational activities until school reopening and help them regulate negative emotions, were largely overlooked. This paper shows that such neglect magnified learning losses during remote learning, by documenting that an intervention targeting these skills successfully mitigated a significant share of those losses among high-school students in Brazil in the aftermath of the COVID-19 pandemic.

We also showed the largest effects of the intervention were concentrated in the upper half of the proficiency distribution, and in schools that already featured online academic activities prior to the pandemic. Together, those findings are consistent with the claim that curricular content and socio-emotional skills are complements for effective learning. They also indicate that effective remote learning activities might be necessary – even if clearly insufficient—to keep *all* students engaged and motivated to return to in-person classes when the conditions allow.

Interestingly, the magnitude of the effect sizes we estimate suggest that interventions focused on providing remote remedial lessons during the pandemic might have at least partly worked by shifting education’s motivational salience (in line with ref. ^21^).^19^, for instance, document that text messages and phone calls with targeted remedial instruction increased test scores in Botswana by 0.12−0.17 s.d. during the pandemic. In our experiment, treatment effects on math standardized test scores lie close to the lower range of that interval, and those on Portuguese, above its upper range—even in the absence of instructional content. While merely engaging students is clearly insufficient—as our treatment effects were concentrated on schools that had previous experience with remote learning—, we show that motivating students when they have (even extremely crude) means to study remotely achieved impacts of the exact same order of magnitude as those of delivering targeted instruction.

All in all, our findings provide important lessons to address the global education crisis in the context of the pandemic. Beyond focusing on curricular knowledge to address learning deficits, public school systems should reach out to families to provide support and encouragement during challenging times. While having school staff adequately trained to handle students’ emotional and psychological needs would be ideal, the limited supply of trained specialists and the budget constraints of public school systems in middle- and low-income countries often make such ideal unattainable. As such, simple technologies such as text messages can help partially achieve that goal, under much lower complexity and costs. At a minimum, that approach can complement the work of school counselors, and ensure that students remain supported even when the latter are not available. Cell phone penetration is very high worldwide^22^ and text messages do not require smartphones or internet access. Despite illiteracy challenges, there is recent evidence that nudges via text messages can work just as well as audio messages^23^. That said, the same might not be true in the absence of in-person classes, especially in low-literacy settings.^19^ document that text messages did not work stand-alone in Botswana during the pandemic (only when accompanied by automated phone calls).^23^ find that illiterate parents in Côte d’Ivoire benefited from text messages because they sought teachers’ help to interpret them (which might not have been possible without in-person classes). Public school systems should carefully select the right technologies to reach out to families remotely in order to effectively provide support and encouragement during challenging times. Above and beyond choosing the right media, they should also pay attention to best practices when it comes to designing and sequencing content, from the frequency of communication (e.g.,^24^ document that three messages a week might be optimal; educational impacts seem to backfire after that, consistent with information overload) to features such as message delivery times and interactivity^21^.

Recent evidence shows that, under in-person classes, behavioral nudges—typically sent through text messages to caregivers’ cell phones—have the potential not only to significantly improve learning outcomes^21,25–29^, but also to drastically decrease student dropouts in developing countries^23^. It is surprising that these effects replicated under remote learning, because such nudges typically work by inducing parents to show up in school more often, in particular to monitor teachers more closely^23^—a mechanism that might no longer be meaningful in the absence of in-person classes. The fact that our effect sizes are greater or equal to those documented in previous studies improves our understanding of why these interventions work under typical conditions. In particular, it rules out that parent-teacher interactions triggered by nudges are a necessary condition for these interventions to improve learning outcomes—at least in a context where literacy is not a critical constraint.

A limitation of our study is that we could not directly measure students’ grit, mindset or self-regulation, given the limitations to surveying students during remote learning. Nevertheless, we discussed evidence from SMS surveys that students’ motivation to return to school by the time in-person classes would resume were significantly affected by the intervention in the short-run. Moreover, we can rule out several alternative explanations for our findings, such as changes in school-wide practices (since improvements are concentrated on the grades targeted by the intervention) or experimenter demand effects from students or teachers who know they are targeted by the intervention (since SMS content, which was partly randomized at the student level—without teachers’ knowledge of it—significantly affected effect sizes).

Another limitation is that our experimental findings are based on the ~40% of students for whom the Education Secretariat had valid phone numbers. This highlights two important concerns. First, what keeps schools from establishing direct contact with the remaining 60% of public-school students in the State? For most of those students whom the Education Secretariat was unable to reach, the problem was not that there were no phone numbers on record, but rather that the listed number was incorrect. It is common among people living in poverty to frequently change phone numbers—among other reasons, to avoid insistent calls from debt collection companies (in a country where 2 out of 3 adults have a negative credit score). Distributing SIM cards with earmarked connectivity, a policy adopted by less than 20% of Brazilian States in the 2020 school year^30^, might have the added benefit of establishing a reliable communication line between schools and low-income parents. Second, to what extent are our findings expected to generalize where that communication barrier could be overcome? Previous research suggests that effect sizes would likely be even larger: socio-emotional nudges tend to work best among students from disadvantaged backgrounds^21^.

More broadly, while we expect that these findings would generalize to other low- and middle-income countries—and even to resource-constrained settings of high-income countries such as the United States—, additional research is needed to understand the extent to which this is the case. Even though in-person classes are now back all over the world, accumulated learning losses are still sizeable, and recent evidence from Brazil suggests that neglecting students’ socio-emotional skills continues to play a role—this time, dramatically holding back the speed of learning recovery^31^. In effect, recent research documents that SMS nudges targeting socio-emotional skills were the most effective intervention to boost learning recovery among a wide range of remedial policies in São Paulo, Brazil, doubling recovery rates relative to the State average^32^. Nonetheless, socio-emotional needs might vary widely across settings, before, during and after the pandemic. As a leading example^33^, document that the relationship between children’s self-perceptions (an important component of mental models encompassed by the broad category of socio-emotional skills) and reading achievement in the 2021 PISA varies significantly across countries with high and low social mobility. More broadly^34^, document that culture and social structure deeply influence the extent to which people are able and willing to regulate their emotions and self-interest in the context of cooperation and reciprocity games. A key finding is that the local importance of cooperation chiefly mediates behavioral differences across settings—from those integrated to markets to foraging societies to nomadic herding groups. In face of those differences, if the impacts of SMS nudges on students’ educational outcomes extend to other settings, then not only would researchers learn about the extent to which our findings can be generalized, but also, policy-makers would have a more solid evidence base to more confidently (and more quickly) incorporate socio-emotional communication with students in their educational policy toolkit. As such, this remains an important avenue for future research.

## Methods

### Socio-emotional skills, learning outcomes, and behavioral nudges

Previous work documents that a range of socio-emotional skills systematically correlate with learning outcomes during “normal times“. As leading examples: (i) students’ intrinsic motivation (and, specifically, challenge-seeking preferences) consistently predicts learning outcomes among third-, fourth- and fifth-graders in the United States^11^; (ii) students’ grit (measured by their time preferences, specifically with respect to their ability to delay gratification) systematically correlates with math and language grades among fourth-graders in Turkey—and, most importantly, an intervention that causally increases grit significantly improves learning outcomes^8^; (iii) students’ self-regulation (assessed through different strategies, from time-use diaries to think-aloud methods) predicts learning outcomes from K-12 to university students in the United States^10^; and (iv) a growth mindset (beliefs that intelligence is not a fixed trait, and that performance can be improved trough effort) correlates with math and science GPA of high-school students in the United States – and, most importantly, an intervention that causally promotes a growth mindset significantly improves learning outcomes^12^).

While different socio-emotional skills are often studied independently, they naturally tend to be positively correlated (also with executive functions; see e.g.,^35^, which documents such correlations for primary students in Côte d’Ivoire). Since that makes it challenging to evaluate impacts on a specific socio-emotional skill and how these impacts trickle down to learning outcomes, separately from other skills, we treat those factors as a bundle throughout the paper—comprising the ones discussed above in addition to others similarly associated with students’ learning outcomes.

That evidence afore-mentioned motivates our main research question: could targeting students’ socio-emotional skills during the pandemic have at least partially mitigated its dramatic negative impacts on math and language proficiency?

The answer to that question is not obvious. On the one hand, motivation, grit, self-regulation and a growth mindset might be even more important without the structure provided by in-person classes and without adequate conditions to focus on academic activities during remote learning. On the other hand, these skills might actually have played a less prominent role during remote learning, in the absence of face-to-face interactions, social pressure and other sources of motivational salience that often detract from learning, especially during adolescence^6^.

This paper studies that question taking advantage of a field experiment in the State of Goiás, Brazil, that randomly assigned a text-message (SMS) nudge intervention across high-school students in the State. Behavioral nudges are interventions intended to modify the choice architecture—i.e., they change the way decisions are framed, in order to mitigate or amplify behavioral biases, inducing certain decisions while preserving subjects’ freedom of choice. These interventions have been shown to effectively change behaviors across various contexts, from preventive health care to savings to education^36^. Refs. ^25^ and^14^ provide great summaries of the existing literature on the use of nudges to influence parental behavior and improve learning outcomes of children. While our experiment focuses, for the most part, on SMS nudges sent directly to students themselves, many of the lessons learnt from the studies discussed in these papers are also applicable in our case. In effect, a number of studies find little difference in the effectiveness of interventions when parents are added to student-only messaging programs (e.g.,^25^).

The existing research suggests that such interventions work by addressing a combination of (1) informational constraints, (2) attentional constraints, and (3) lack of future orientation. We discuss these potential mechanisms in detail in the Supplementary Materials. For our purposes, it suffices that the intervention increases students’ socio-emotional skills—be it by inducing students to update beliefs, by redirecting their attention, by changing their preferences directly, or through a combination of the above (or even through alternative mechanisms that the literature has not focused on).

### Hypotheses

Concretely, we test the following hypotheses:

1. [Outcomes] Did the SMS nudge intervention *decrease* math and Portuguese learning losses, relative to the control group?
2. [Mechanisms] Did the SMS nudge intervention *increase* student attendance and motivation to go back to school once in-person classes returned?

Hypothesis 1 is informative about whether the intervention was effective in averting at least part of learning losses in the context of remote learning. If the evidence backs it up, it is consistent with the hypothesis that neglecting students’ socio-emotional skills magnified learning losses during the pandemic. If we find confirmatory evidence for hypothesis 1, hypothesis 2 is informative about the mechanisms underlying such impacts. Concretely, we test whether the intervention indeed boosted student’s socio-emotional skills (constrained by the ones we could measure in the absence of in-person classes).

One concern with the above is that, since the SMS intervention was randomized at the school level, treatment effects could be driven at least partly by alternative mechanisms: students might have been reminded of school activities, or inferred school norms about effort from the messages (even if they did not explicitly conveyed such content); or teachers might have increased effort because they knew their school was being targeted by an intervention (regardless of its content), relative to control schools.

To rule out that concern, we additionally randomized the intervention script *at the student level*. This allows us to test the following additional hypothesis:

(3) [Robustness] Were the effects of different SMS interventions on learning losses *different*?

Hypothesis 3 is informative about whether the content of the intervention ultimately matters, which is key to rule out those alternative explanations. The reason is that while the school was aware of overall treatment status, it was not informed about which student was being targeted by which set of messages.

These hypotheses were pre-registered as part of a pre-analysis (trial 5986 at the AEA RCT Registry, included in the Supplementary Materials).

### Background

In Goiás, the setting of our study, in-person classes were suspended in March 2020, and did not resume until August 2021. During school closures, classes transitioned to online, delivered through a videoconferencing and team collaboration platform. Students were assigned daily exercises that they had to hand in through the platform. For those without internet access, schools handed out assignments in plastic bags hung at the school gate, and students had to hand them back in the same way. Those patters were largely representative of Brazilian public schools during the pandemic, and the State’s response was also rated around the national median^30^.

Our study restricts attention to full-time high-schools, whose students stood for nearly 50% of all high-school enrolled in the State by 2020. As in many other developing countries, Brazilian public schools rely on multiple shifts to accommodate the large number of students seeking enrollment under its limited infrastructure: schools typically feature two sets of students, spread through its morning and afternoon shifts (in some schools, there is also a night shift). In these schools, students spend 4.5 h in school each weekday. Over the last decade, in response to the slow progress in learning outcomes, particularly among high-school students, Brazilian States have started offering full-time high school programs, with extended hours of teaching. In these programs, students are in the classroom 8 h each weekday. Full-time high school programs feature similar core curricula as regular schools. Recent evidence suggests that such programs have positive effects on student test scores^37^. Relevant to the generalizability of our study, student characteristics do not differ systematically across part-time and full-time high-school programs^37^.

### Research design and intervention

To study the effect of behavioral nudges on educational outcomes during remote learning due to the pandemic, we conducted a cluster-randomized control trial in the State of Goiás, Brazil (pre-registered as trial 5986 at the AEA RCT Registry and included in the Supplementary Materials). This was carried out in partnership with Instituto Sonho Grande and Goiás State Secretariat of Education in the context of their full-time high school program (*Ensino Médio em Tempo Integral*) and approved by the Institutional Review Board of the Department of Economics at the University of Zurich (2020-033).

The intervention, powered by Movva, consisted of sending behavioral nudges twice a week over text messages (SMS) to public school students enrolled in grades 10−12, typically aged 15−18 years old, or their primary caregivers. Nudges consisted of encouragement messages sent twice a week, organized in thematic sequences of four messages, with a new sequence starting every other week. Nudges targeted students’ socio-emotional skills; in particular, messages tried to motivate students to stay engaged with school activities during remote learning, to support them in regulating negative emotions, to foster a growth mindset, and to develop grit; examples are provided in Supplementary Materials. The intervention evaluated in this study has been shown to improve educational outcomes in the context of in-person classes across different settings^21,23^.

The intervention spanned the universe of State schools offering the full-time high-school program. The Education Secretariat had access to valid phone numbers for 18,256 students, roughly 40% of the total. Since participants are minors, broad consent was obtained from their legal guardians directly by the Education Secretariat (at the time of school enrollment), allowing researchers to use secondary information from administrative records without eliciting further consent. Our implementing partner Movva further obtained students’ assent directly via text messages (SMS): participants were informed and reminded of the fact that they could opt-out of the intervention and SMS surveys at any point (by simply replying “STOP” or “CANCEL”, free of charge), without consequence. While we do not have data on students outside our sample, studies in other Brazilian States provide insight into the nature of selection: students whose phone numbers are known by the school tend to be from wealthier households and display higher grades^21^. We discuss the implications of selection to the generalizability of our findings in the “Discussion” section.

In total, 12,056 high-school students across 57 public schools received SMS nudges, while 6200 high-school students across 30 public schools received no nudges or other text messages from their schools over that period (see State map in Supplementary Materials). Randomization was undertaken at the school level, stratified by gender, grade and phone ownership. 42% of treated students received nudges directly on their phones, whereas 58% had nudges targeted at their primary caregivers’ phones—this was not randomized; rather, it happened only when the Secretariat did not have access to students’ phone numbers directly. The intervention started on June 9, 2020 (during Q2/2020), and continued until July 7, 2021 (at the end of Q2/2021). No messages were sent during the winter break in July, 2020. Only 1.14% of participants opted out over the course of the study.

More students were assigned to treatment than control because we split treated students into additional treatment arms, varying the content of nudges across them. We cross-randomized treated students to (1) a framing experiment, and (2) a social pressure experiment. Randomization was undertaken at the individual level. These additional experiments took place in the first month of the intervention, during Q2/2020. All treated students received exactly the same content from Q3/2020 until the end of the intervention. In the framing experiment, half of treated students were assigned to a message framing the prospects of staying in school in terms of gains (the upside of high-school completion), and the other half, to a message framing these prospects in terms of losses (the downside of school dropouts). In the social pressure experiment, half of treated students were assigned to a message stating that 80% of their fellow students wanted to return to in-person classes after school reopening (based on results of an SMS survey), and half to a message that just stated the importance of returning to in-person classes without reference to or data on peers’ motivation to do so (see Supplementary Materials).

Differences in effect sizes across treatment arms of the content experiments help us rule out that the effects of nudges were merely driven by experimenter demand effects (e.g., if students merely reacted to the salience of school activities or inferred social expectations about school effort upon being targeted by the intervention). Moreover, while teachers were aware of the school-level assignment, they were unaware of student-level content variations, which were randomly assigned and implemented directly by Movva over a limited number of weeks early on in the intervention. As such, the content experiments also help us rule out that treatment effects merely reflected teachers’ strategic responses (e.g., in case they exert higher effort in treated schools relative to the control group).

### Ethics approval and consent

Approval for this study was obtained from the Institutional Review Board of the Department of Economics at the University of Zurich (2020-033). Additional ethics approval by a local IRB is waived in Brazil for research that is not health related. When it comes to informed consent, since participants are minors, broad consent was obtained from their legal guardians directly by the Education Secretariat (at the time of school enrollment), allowing researchers to use secondary information from administrative records without eliciting further consent. Additional parental consent for access to student data or for participation in the SMS intervention or in the SMS surveys was waived by the Education Secretariat, based on minimal risk to study participants. Our implementing partner further obtained students’ assent directly via text messages (SMS): participants were informed and reminded of the fact that they could opt-out from the intervention and SMS surveys at any point (by simply replying “STOP” or “CANCEL”, free of charge), without consequence.

### Definition of outcomes and estimation

#### School-level proficiency

We start by estimating treatment effects of nudges on school-level proficiency levels in the 2021 State-wide standardized exam. We estimate treatment effects of nudges on average proficiency levels, pooled and separately by Portuguese and math, and on the share of sub-proficient schools (those below basic proficiency levels, according to the State Secretariat’s definition; https://avaliacaoemonitoramentogoias.caeddigital.net/resources/arquivos/colecoes/2019/GO%20SAEGO%202019%20RG%20WEB.pdf). In this dataset, each observation is a 4-tuple school-grade-subject-year. Proficiency ranges from 0 to 500 within each subject. An average test score of 250 or above indicates basic proficiency or higher. As such, we define a sub-proficient indicator equal to 1 if a school’s average score in that subject is below 250 in that year, and 0 otherwise.

We have 2021 data on school-level Portuguese and math proficiency levels for high-school seniors in 58 out of the 87 schools that are part of our experiment. The reason for missing data is that the State does not disclose school-level proficiency levels when less than 80% of the students in a school take the exam. 1 of these schools had no data for 2019, and 3 of them, no data for 2018. As such, we have 170 observations for each subject for the 2018–2021 period, and 340 when we pool math and Portuguese proficiency levels in our analyses. Out of these 58 high schools, 38 also had data for 9th-graders’ proficiency levels in 2021 (27 in 2019 and 29 in 2018), which we use to estimate placebo effects. Pooling math and Portuguese proficiency levels, we have 188 observations in that case. Last, when we pool data for high-school seniors and 9th-graders in the analyses of treatment and placebo effects on the share of sub-proficient schools across both subjects, we have 528 observations in total.

Estimating treatment effects on school-level proficiency levels is a useful starting point for three main reasons. First, standardized proficiency levels are available since 2018 (except for 2020, when in-person exams could not be conducted), allowing us to benchmark the effect size of nudges relative to different counterfactual scenarios, and estimate their quantitative contribution to mitigating learning losses during the pandemic. Second, drawing on these data minimizes concerns with experimenter demand effects (since the exam was not tied to the intervention in any way) and with potential conflict of interest (since the entire history of school-level proficiency data is publicly available). Third, data on proficiency levels for previous years and earlier grades within each school allow us to estimate placebo experiments, to gauge whether treated schools were already improving, or whether they improved in 2021 even for grades not targeted by the intervention.

#### Student-level standardized test scores

Despite these advantages, school-level data hurt the precision of estimated treatment effects on proficiency levels. What is more, if nudges affect the composition of students across treated and control schools (e.g., because of student dropouts), then effects on learning losses could at least partly conflate selection effects. For these reasons, we next evaluate the impacts of nudges on learning outcomes by taking advantage of student-level standardized test scores from an in-person assessment conducted in Q2/2021 (between April and May) with a random sample of full-time high-school seniors in the State.

This additional exam was commissioned by Instituto Sonho Grande. We have access to data on Portuguese test scores for 1337 of our study participants and on math test scores for 1336 of these students (1321 of whom were junior students in the previous year, and the remaining 15, seniors who repeated the grade). Of these students, 453 belong to the control group, and the remaining 883, to the treatment group, split between the framing and social norms experiments. Test scores are normalized relative to the control group mean (such that the control group mean is 0 with standard deviation 1). Following, we also compute a summary measure to deal with family-wise error rates in multiple hypotheses testing. We document that standardized test scores in this additional exam are not subject to selective non-response across treatment conditions. Descriptive statistics are presented in Supplementary Materials.

#### Student motivation and attendance

To provide additional evidence about the mechanisms underscoring treatment effects on learning outcomes, we rely on administrative data on students’ daily attendance in the two weeks before the winter break—shortly after the intervention was rolled out—, and on survey data on students’ motivation to return to school once they reopen, based on self-reports. We elicited the latter weekly over text messages, from rotating sub-samples of students in the treatment and control groups, from the week after the intervention started until 3 weeks into the winter break. These surveys targeted random sub-samples of around 15% of our sample each week, with an average response rate of 13% across weeks. Characteristics of respondents are balanced across the treatment and control groups, and we account for selective non-response in any particular week by appropriately bounding our estimates of treatment effects. We compile results in Supplementary Materials.

#### Additional outcomes

Supplementary Materials show that the intervention did not affect take-up of standardized tests, ruling out that the effects are driven by selective attrition driven by the intervention.

In a companion paper^16^, we document that the intervention also did not systematically affect grade repetition or student dropouts on average, although it did significantly reduce dropout risk among students at the highest risk of leaving school.

### Estimation

We estimate average treatment effects on these outcomes with Ordinary Least Squares (OLS) regressions. Since we cannot verify whether students effectively read the content, we estimate intention-to-treat (ITT) effects based on treatment assignment.

For school-level proficiency, since we have pre-treatment data, we estimate a differences-in-differences model with school fixed-effects.

Since nudges were randomized across schools, we cluster standard errors at the school level, except when we estimate the effects of the content experiments—as those were randomized *within* treated schools, at the student level. When we analyze the treatment effects of the latter, we also include classroom fixed-effects.

We also estimate heterogeneous treatment effects by school’ proficiency level in the 2019 State-wide exam, by students’ first quarter (Q1) GPA, gender and grade, by whether schools already offered online academic activities before the pandemic (according to the 2019 Brazilian School Census), and by whether students’ or caregivers’ phones were targeted by the intervention.

### Statistical power

Given the relatively small number of clusters (J=87), our experiment is powered only to detect large educational impacts. With 1337 observations for student-level standardized test scores, 2/3 of the observations assigned to the treatment group, and an intra-cluster correlation of 0.117 in the case of our student-level Portuguese standardized test scores (see Supplementary Materials), only estimates of 0.2 s.d. or above could be detected as statistically significant (one-tailed test at the 10% level) without controls in the preregistered specification. Since effect sizes of behavioral nudges on test scores are often in the 0.08−0.09 s.d. range^14^, the experiment design is underpowered to detect relevant treatment effects. Whenever our estimates are greater or equal to those magnitudes but not statistically significant, we refer to them as imprecisely estimated.

### Balance and selective attrition tests

Supplementary Materials document that treatment assignment was balanced across student and school baseline characteristics.

When it comes to selective non-response, the additional standardized test conducted in Q2/2021 has missing scores for roughly 35% of students who were randomly drawn to take it. According to Instituto Sonho Grande, such high incidence of missing scores reflects a combination of different factors: students who could not be contacted to take the exam without in-person classes, those who were successfully contacted but who refused to participate, those who agreed to participate but did not show up, and those who showed up but did not attempt a single question. Supplementary Materials show that such high non-response rates are not a concern for our analyses of treatment effects: there are no significant differences across the treatment and control groups when it comes to the share of students with missing standardized test scores. In any case, we assess the sensitivity of our findings to re-weighting observations by the inverse of their predicted probability of taking the exam (estimated through a simple logit regression based on student and school characteristics) to ensure that effect sizes are representative of the universe of students in full-time high schools in the State.

### Reporting summary

Further information on research design is available in the Nature Research Reporting Summary linked to this article.

### Supplementary information

Supplementary Materials

Reporting summary

## Data Availability

The data that support the findings of this study (without PIIs) are available at https://osf.io/3sqfr/.

## Code availability

A complete replication package is available at https://osf.io/3sqfr/.
