# Supplementary material for: Neglecting students’ socio-emotional skills magnified learning losses during the pandemic
Source: NPJ Sci Learn. 2024 Apr 9;9:28. doi: 10.1038/s41539-024-00235-9 (PMC11003960; doi:10.1038/s41539-024-00235-9)
Supplement: Supplementary file 1 — Supplementary Materials [file 41539_2024_235_MOESM1_ESM.pdf]

Part

Supplementary Information

Table of Contents

A Supplementary Methods **37**

A.1 Additional Information on the Experiment 37

A.2 Literature review 45

A.3 Pre-Analysis Plan 49

B Supplementary Figures and Tables **56**

B.1 Descriptive Statistics 56

B.2 Balance and Selective Attrition Tests 60

B.3 Short-Run Effects on students' Motivation and Attendance 66

C Supplementary References **71**

A Supplementary Methods

A.1 Additional Information on the Experiment

Participants. Participants consist of public school students enrolled in grades 10-12; typical age is 15-18 years old. All contacts were provided to Movva by the Goiás State Secretariat of Education. The total number of contacts in the database correspond to 18,256 students, 12,056 of which randomly assigned, across 57 schools, to receive SMS nudges between June 9th and December 31st, and 6,200 across 30 schools assigned not to receive nudges or any other SMS communication from their schools. Power calculations before the onset of the intervention pointed out this sample size was large enough to detect relevant minimum effects on the outcomes of interest.

Data collection. Before the start of the intervention, the contacts' database was shared with the authors to complete the randomization at the school level, stratified by gender, grade and phone ownership. Schools were randomly assigned to either a treatment or a control group, following the group sizes above and using the statistical software Stata. The database including a treatment assignment indicator was then returned to Movva such that SMS nudges could be sent accordingly to the treatment group, but not to the control group. Data on online access to the platform and participation in offline school activities was shared by the Secretariat of Education with Movva, while data on motivation to return to regular classes once they resume was collected by Movva directly over SMS surveys, from rotating sub-samples of approximately 280 students in the treatment and control groups every week – from the week after the intervention started until 3 weeks after it ended. Weekly sub-samples were also randomly drawn from the subject pool. Data on quarterly grades and attendance was shared by the Secretariat of Education in March 2021. Data on actual test scores in Portuguese and math from an assessment test conducted in

person between April and May 2021 was shared by the Secretariat of Education in July 2021. Balance tests using Wald tests of simple and composite linear hypotheses were conducted before and after propensity score re-weighting to ensure that each treatment group is comparable with respect to students' and school characteristics. These tests and results are detailed in Appendix B.2. Outcome data was shared with the authors, and analyzed following a pre-analysis plan pre-registered as trial 5986 at the AEA RCT Registry (Appendix A.3). We did not pre-register that we would analyze treatment effects on the high dropout risk proxy rather than official enrolment status because we did not anticipate at the time that the State would automatically enroll all students in 2021. We also did not pre-register the additional experiments, varying content within treated students, that we report in a separate section. All analyses were conducted by the authors using the statistical software Stata. Finally, collecting information on human participants over time is subject to attrition. Participants were free to leave the study at any time, which creates a risk of biasing the results if such attrition is correlated with treatment assignment. In this context, we tested whether the probability of students responding to the SMS surveys was affected by the treatment. The results, which are reported in appendix B.2, indicate that the probability of responding to SMS surveys is not systematically affected by the treatment.

Intervention. Movva, the start-up that powered the intervention evaluated in this study, specializes in promoting behavior change by sending nudges – frequent reminders and encouragement messages – directly to users' cell phones. Eduq+, the intervention evaluated in this study, has been shown to improve educational outcomes in an environment of regular classes across different settings (Lichand et al., 2022; Lichand & Wolf, 2021). In the context of this study, two nudges per week were sent over text messages (SMS) to high-school students or their primary caregivers,

depending on phone ownership, in the treatment group. Nudges were organized in 2-week sequences of 4 messages, as follows (translated from Portuguese):

Supplementary Table 1
Sample SMS sequences

	Week 1		Week 2	
Treatment	Fact	Activity	Interactivity	Growth
Self-regulation	Distractions com come from FEELINGS such as boredom, insecurity and anxiety. IDENTIFYING what distracts us is the first step to be able to FOCUS.	Note down TODAY what distracts you from studying. Thing about what CAUSES these distractions and about HOW TO DEAL with them: WHEN [CAUSE], I WILL [PLAN TO ADDRESS IT].	Were you able to identify what DISTRACTS you? Tell us you main source of distraction and EARN a technique to boost your FOCUS (answer free of charge).	To help you come up with a plan, here is an example: "WHEN I study next to my phone, I WILL turn off push notifications!". What are your strategies?
Growth mindset	A GROWTH MINDSET means believing we can always IMPROVE relative to ourselves: the more we TRY, the BETTER we get.	RESEARCH the story of someone you admire, like a great athlete or artist. Identify FAILURES and how s/he had to PERSIST TO OVERCOME THEM.	We want to know: do you think your INTELLIGENCE and SKILLS are fixed and cannot be changed? Answer free of charge to get a tip.	Students who have a GROWTH MINDSET have better outcomes, in HIGH SCHOOL and in the FUTURE. Focus on the motto: the more you PRACTICE, the BETTER you get!
Motivation	Last mile! This was an intense year, but you got here! ø/ Let's take a deep breath and reach the finish line together AlmostThere!	Just a few more steps! Talk to your school TODAY and figure out what you have to do to move on to the next grade. Make a LIST not to forget anything!	To make it happen, you mind has to be in the mood! Are you CONFIDENT that you can progress to the next grade? Answer YES or NO (free of charge).	That's the spirit! With a motivated mindset, you can put together what it takes to join the GRADE PROMOTION wagon! Hang in there!
Resilience	2020 has been tough! But we will remembered it for not having given up on our dreams! Assessing what we have learned and revising our plans helps us stick to them!	To plan our future, we need to look back first. Grab a piece of paper and note down what life lessons 2020 brought. Challenges can teach valuable lessons!	Challenges require adaptations. How motivated are you to incorporate the lessons learned to your LIFE PROJECT? 1- Highly, 2- Moderately, 3- Not very (answer free of change).	Great! It is important to always come back to your LIFE PROJECT. HangInThere and focus on your studies to achieve your dreams!

Supplementary Table 2
Content experiments (Q2/2020)

	Week 1		Week 2	
Treatment	Fact	Activity	Interactivity	Growth
Framing losses	It is normal to be afraid in times of uncertainty. Use this to your favor: take the opportunity to develop your ability to focus on future plans.	How about reviewing your "Projeto de Vida"? Highlight which dreams you would regret not accomplishing. Plan each step to be successful.	Tell us! From 0 to 10, what is your level of confidence that finishing high school will help your future plans? SMS free of charge.	Never forget: your future depends on your studies. Focus on activities NOT TO LOSE your high-school degree and what you have achieved so far!
Framing gains	It is normal to be afraid in times of uncertainty. Use this to your favor: take the opportunity to develop your ability to focus on future plans	How about reviewing your "Projeto de Vida"? Highlight which dreams you would regret not accomplishing. Plan each step to be successful.	Tell us! From 0 to 10, what is your level of confidence that finishing high school will help your future plans? SMS free of charge.	Never forget: your future depends on your studies. Focus on activities TO ACHIEVE your high-school degree!
Social pressure	Be aware! 80% of your fellow students believe that finishing high school can lead to a good future. To get there, you must organize your study time!	Set up a schedule for the day: set a time to wake up, to study, to do activities and, of course, to watch the latest episodes of your favorite show.	Using a full day to study leaves you closer to your degree. How has your time management been? 1.Good 2.Regular 3.Bad. SMS free of charge.	Well-deserved holidays! Time to rest, but without losing the focus on the future. Between leisure activities, find something that you like and that helps you learn.
No social pressure	Be aware! The high school degree opens many doors to your future. To get there, you must organize your study time!	Set up a schedule for the day: set a time to wake up, to study, to do activities and, of course, to watch the latest episodes of your favorite show.	Using a full day to study leaves you closer to your degree. How has your time management been? 1.Good 2.Regular 3.Bad. SMS free of charge.	Well-deserved holidays! Time to rest, but without losing the focus on the future. Between leisure activities, find something that you like and that helps you learn.

Measures. *School-level proficiency levels in State-wide exams:* Publicly available data on school-level proficiency in the State-widge standardized exam for 2018, 2019 and 2021. This dataset contains average proficiency by school, grade (5th, 9th and 12th), subject (math and Portuguese), and year. Proficiency ranges from 0 to 500 within each subject. An average test score of 250 or above indicates basic proficiency or higher. As such, we define a sub-proficient indicator equal to 1 if a school's average score in that subject is below 250 in that year, and 0 otherwise.

Student-level standardized test scores: Instituto Sonho Grande shared administrative records on standardized test scores with the authors. This dataset contains information on actual test scores for 2,044 of our study participants from an assessment test conducted in person between April and May (in the second school quarter) 2021. Math and Portuguese test scores were available for 1,336 out of those students. We standardized scores withing each subject relative to the control group (such that test scores in our analysis have a mean of 0 and standard deviation of 1). We also generated a summary measure defined by the arithmetic mean of these two scores to deal with family-wise error rates in multiple hypotheses testing following Kling et al. (2007).

Student's prolonged absenteeism right before the winter break: The Secretariat of Education shared administrative records with Movva, which then shared it with the authors. This dataset contained information on access to the online platform and participation in offline school activities at the student level, an indicator variable equal to 0 if the student logged in to the platform or participated in offline school activities on a given day and 1 in case they did not. Based on this information, we created a measure of prolonged absenteeism which was equal to 1 if a student had no attendance on record for two weeks in a row right before the winter break (between June 15th and June 26th), and 0 otherwise. See Appendix B.3.

Student's motivation to return to school once they reopen: Each week, students assigned to be surveyed by text message reported their motivation to return to school once regular classes resume by answering the following question: "Do you plan on returning to school once regular classes resume?". Movva coded lack of motivation to return as a binary indicator based on SMS replies, equal to 1 if the reply was “No” or similar, and zero otherwise, and shared that information with the authors. Student dropout risk. We define high dropout risk equal to 1 if a student had no math and no Portuguese grades on record in that school quarter, and 0 otherwise. See Appendix B.3.

Analysis method. All results presented in the paper use intention-to-treat analyses by linking student identification numbers to the treatment condition they were assigned to before the start of the intervention. The Education Secretariat did not share information on siblings, so we could not drop pairs assigned to different treatment arms as we had specified in the pre-analysis plan. This is not an identification threat since within-household spillovers would only lead us to under-estimate treatment effects. Throughout the paper, we report intention-to-treat effects obtained from Ordinary Least Squares (OLS) regressions, by regressing each outcome on a binary indicator equal to 1 if the student was assigned to treatment and 0 otherwise. We restrict our analyses to intention-to-treat (ITT) effects because that we had no means of verifying whether students effectively received messages as intended. All p-values comparing treatment and control groups are obtained from t-tests of equality of coefficients between the treatment and control groups, with standard errors clustered at the school level in each case. All p-values comparing groups assigned to the content experiments within the treated group are obtained from t-tests of equality of coefficients between these treated groups, with classroom fixed-effects and standard errors clustered at the individual level in each case.

Supplementary Figure 1

Spatial distribution of treated and control schools

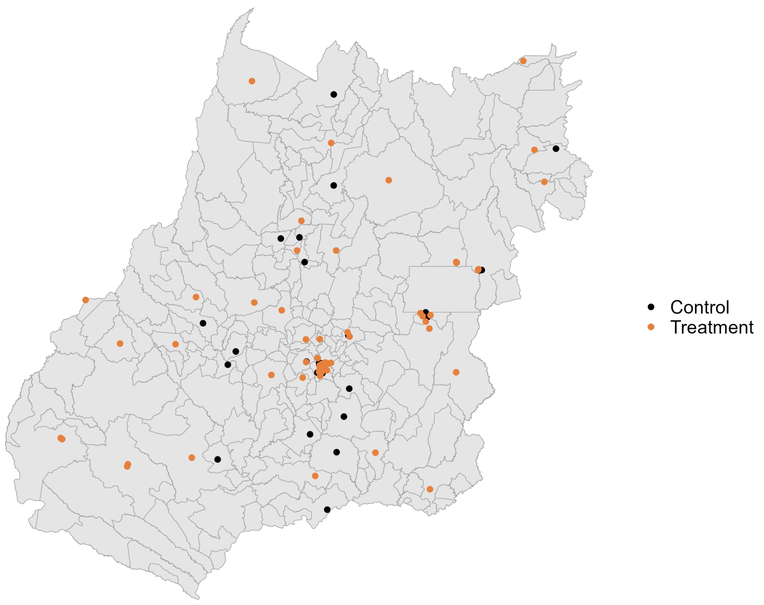

Notes: Map of Goiás State, showcasing treated (orange) and control (blue) schools.

A.2 Literature review

This section outlines alternative mechanisms through which behavioral nudges might affect learning outcomes amongst children and adolescents.

Information

A number of studies have shown parents to have inaccurate information about their children's school effort and performance, as well as about the future returns to education in terms of expected employability and wages. In particular, parents tend to overestimate their children's school attendance (e.g., Bergman, 2021; Lichand et al., 2022) and school ranking (e.g., Dizon-Ross, 2019), and underestimate the returns to schooling (e.g., Jensen, 2010).

In face of informational constraints, providing parents with accurate information on their children's performance relative to their peers can lead to higher monitoring (Bergman, 2021), improving educational outcomes (e.g., Siebert et al., 2018; Barrera-Osorio et al., 2020; Dizon-Ross, 2019).

Naturally, students themselves can be systematically inaccurate in their self-appraisal of academic performance, or in their expectations about returns to effort (e.g., Yeager et al., 2019). As such, nudges to students may also help them to overcome informational constraints, increase school effort and improve educational outcomes.

Attention

Perhaps surprisingly, recent evidence suggests that nudges can lead to the same improvements even without child-specific information, by inducing parents to acquire information independently about their children's school performance (Lichand et al., 2022).

This evidence lines up with the hypothesis that executive functions (including attention, working memory and self-control) are constrained by the availability of mental bandwidth, which is taxed by financial worries linked to poverty (Mullainathan and Shafir, 2013). A number of studies have found poverty to reduce cognitive capacity precisely because of the drain that financial instability imposes on mental resources, leaving less bandwidth available for other decisions and tasks (e.g., Mani et al. 2013; Gennetian and Shafir 2015). Ultimately, under such stressors, sub-optimal decisions are likely to emerge, particularly when it comes to investments in children's education (Bergman, 2019).

In this context, nudges can reallocate parents' attention to children's school life. Lichand et al. (2022) show that even though nudged parents do not become more accurate about their children's attendance, they do become more accurate about the extent to which their children's GPA changes over the course of the school year, albeit only coarsely – consistent with higher monitoring effort.

When students are nudged directly, similar mechanisms might be at play: nudges might encourage them to acquire information about their own standing or about returns to effort even if the intervention does not convey information directly relevant to the specific circumstances of the recipient.

Future Orientation

These arguments are tied closely with another strand of the literature which argues that such behavioral nudges are effective in improving educational outcomes by helping participants overcome cognitive biases, in particular when it comes to time discounting. This problem has been well explored in decision-making scenarios in education and investments in human capital (e.g., Sutter et al. 2013; Castillo et al.

2011, Castleman and Page 2014). The tendency to overvalue immediate outcomes relative to future ones can be associated with self-control problems and lead to under-investments in general, in particular when it comes to children's education.

The literature provides a great deal of support for the hypothesis that behavioral interventions such as reminders, goal setting and social rewards can help individuals to overcome present-bias. Mayer et al. (2019) documents that behavioral nudges aimed at increasing parental engagement with their children's education have larger effects among present-oriented participants, consistent with the idea that nudges might also increase future orientation.

It is easy to imagine that such cognitive biases also plague the decision making of children hyperlinkalan2019(e.g., Alan, Boneva and Ertac, 2019). As such, by re-focusing students' attention to long-term goals, nudges might help them to overcome procrastination and other behaviors detrimental to learning.

From Constraints to Behavior

If nudges help students and their families overcome constraints arising from informational and psychological barriers, then they are expected to help engage students more effectively with the educational process (e.g., through higher attendance and homework completion; e.g., Lichand et al., 2022), resulting in improved learning outcomes (captured through higher test scores and lower grade repetition rates; e.g., Lichand et al., 2022) and increased motivation to stay in school (captured through lower dropout rates; e.g. Lichand & Wolf, 2021).

Whether parent-teacher interactions are a key mediator for translating less binding constraints into behavior change is an open question. Lichand & Wolf (2021) illustrate that the nature of these interactions might be key for nudges to ultimately improve

educational outcomes. As such, without these interactions in the context of remote learning (especially in our study setting, where connectivity is very limited, such that, for most families, in-person interactions are not expected to be replaced by virtual interactions), it is not clear that nudges would effectively mitigate learning losses. This is the research question that this paper sets out to answer.

A.3 Pre-Analysis Plan

This randomized controlled trial was pre-registered as trial 5986 at the American Economic Association's registry for randomized controlled trial (AEA RCT Registry). The uploaded plan is detailed below.

Does Nudging Students Decrease Learning Deficits and Dropouts During and After a Pandemic? Experimental Evidence from COVID-19 Responses in Brazil

Pre-analysis Plan

The COVID-19 pandemic has forced 1.5 billion schoolchildren in 160 countries to stay at home while schools were shut down on sanitary grounds. While several remote learning tools have been put in place in developing countries, a variety of factors raise critical concerns about learning deficits and school dropouts when schools are back, particularly amongst the most vulnerable students. This paper investigates whether sending reminders and encouragement messages to high-school students in Brazil during the pandemic increases attendance and assignment completion when it comes to remote learning, and decreases grade repetition and dropout rates in the aftermath.

Introduction

The COVID-19 pandemic has forced 1.5 billion schoolchildren in 160 countries to stay at home while schools were shut down on sanitary grounds. Brazil is no exception. The nationwide decision to shut down schools for almost the entirety of the 2020 school year in order to limit the spread of the COVID-19 pandemic has forced all schools to switch to remote learning. Such rapid transition, combined with a mismatch between delivery channels and access conditions – as several State Sec-

retariats of Education switched to online, while nearly 70 million households have no or only precarious access to internet –, are expected to severely impact learning, and potentially lead to a spike in school dropouts (Vegas, 2020); World Bank, 2020).

Schools have been trying to keep contact with their students by sending personal letters via post or by creating an online platform with tools that students can use. However, the attendance of the students, whether on the platform with the online tools or at school to pick up printed class material, is reported to be remarkably low. São Paulo State has reported that only 50% of its 3.5 million students are accessing the online learning platform daily as expected. The State Secretariat also broadcasts content on television. It is much harder to gather data on the share of students following classes on this format daily.

With the goal of increasing engagement in remote learning – and, particularly, online attendance and assignment completion – during the pandemic, as well as limiting its effects on learning gaps and school dropouts once schools are back, the Goiás State Secretariat of Education is testing various strategies in partnership with Instituto Sonho Grande. Goiás a relatively poor state located in the Center-West region of Brazil. Instituto Sonho Grande is a non-profit organization committed to improving high-school educational outcomes in Brazilian public school. As part of those strategies, they are interested in evaluating nudges (reminders and encouragement messages) sent twice a week to high school students, directly on their mobile phones via text messages (SMS). Towards that goal, they have hired Eduq+, an educational nudgebot that has been shown to improve educational outcomes (during normal times) in Brazil and Ivory Coast.

Eduq+ nudges users twice a week with motivating facts and suggested activities to engage them in the daily school life. It also allows schools to broadcast messages to all users weekly. The intervention has been evaluated in the context of regular

schooling, targeted at parents of primary school children. The nudgebot has been shown to promote large impacts on school attendance, test scores and grade promotion rates (Bettinger et al., 2020), and to decrease school dropouts by 50% across multiple primary grades (Lichand and Wolf, 2020).

The version of Eduq+ to be evaluated in this study is, however, different from that in those studies, since nudges will be sent directly to students themselves.In case they do not have their own phone, messages will be sent to the mobile phone of their primary caregivers. Moreover, the context of remote learning is also much more challenging. Whether the intervention is still able to improve educational outcomes under those conditions is an empirical question.

This pre-analysis plan summarizes the design of a field experiment to test the following primary hypotheses:

- Does nudging students increase usage of online learning tools by high school students?
  - Hypothesis: SMS nudges increase the share of students who access the online platform daily, and the share of students who hand in assignments (online or not).
- Does nudging students mitigate the negative effects of school closures on learning outcomes?
  - Hypothesis: SMS nudges improve attendance and grades, and decrease grade repetition and dropouts once in-person classes resume.

Intervention and experimental design

The intervention has been designed by Instituto Sonho Grande and the Goiás State Secretariat of Education, with the help of Movva (the implementing partner that powers Eduq+). One of the authors (G.L.) is a co-founder and chairman at Movva. It will take place during the months of June and July/2020, when public high schools will be randomly assigned to have their students receive two messages per week from Eduq+. 57 schools have been assigned to the treatment group, and 30 to the control group (which receives no intervention). Randomization is stratified by gender, grade and phone ownership. In case the student does not own a phone, messages will be sent to the mobile phone of their primary caregiver. The intervention is scheduled to be rolled out on June 9th.

Supplementary Table 3
Randomization strategy - Treatment vs. Control

**Treatment**	**Control**
57 schools	30 schools
12, 056 students	6, 200 students

Table 3 above summarizes the randomization strategy for the first phase of intervention. Within the sample of 12,056 students assigned to receive nudges, less than half (5, 188) own their own mobile phone and will receive messages directly. It is also important to note that not all students in the sample have access to the internet and that those who do not can pick up the printed class material once every week and hand in assignments the following week. For the purpose of this study however, we will be able to measure their outcomes in different ways.

At the end of July, we will be able estimate treatment effects on access to the online

platform, and assignment completion, from administrative data provided by the Secretariat. Concretely, we have requested weekly student-level data on log in activity – or face-to-face pick-up of class materials – as well as assignment completion (again, online or offline). For those with online access, we hope to get access to daily data, which would allow us to also estimate high-frequency treatment effects through event studies. Last, after in-person classes resume, we will have access to administrative records on student-level attendance, grades, grade repetition and enrollment status.

The interpretation of these long-term effects will vary depending on the choice made by the Education Secretariat to continue or not the intervention after short-term results are made available. Depending on the short-term impacts of the nudges, the Education Secretariat might decide to keep testing Eduq+ for a longer period, to scale it up or to scale it down. As such, three scenarios can emerge after the first phase of the intervention: (1) the intervention continues for a longer period, keeping the treatment assignment fixed; (2) the control group starts receiving the nudges; or (3) the treatment group stops receiving the nudges. In case (1), long-term effects will reflect a combination of nudges sent during and after school closures; in case (2), long-term effects will only reflect differences in the intensity of the treatment; and in case (3), long-term effects will capture persistence of treatment effects (if any).

With the number of schools and the number of students presented in Table 1, and assuming an intra-cluster correlation of 0.16 (SARESP, 2014a, 2014b) as well as conservative variance estimation for binary outcomes (assuming that 50% of students access the online platform and hand in assignments, in the control group)-, we could detect treatment effects of at least 0.8 percentage points on those outcomes. These power calculations have been computed by clustering at the school level. Since the typical treatment effect of nudges on binary decisions is 1.7 percentage points (Dellavigna

and Linos, 2020), we conclude that the design is well powered to detect relevant short-term effect sizes.

Outcomes

We will document the effects of the treatments on the following categories of outcomes for students enrolled in high school (age 15 to 18):

- Short-term outcomes: probability of logging into the online platform or picking up the material in school, probability of handed in of assignments, as measured by administrative records;

- Long-term outcomes: attendance, grades, probability of grade repetition and probability of dropout, as measured by administrative records.

Since some students will receive messages on their own mobile phones, while for others it is their caregivers who will be nudged by Eduq+, we will estimate treatment effects within those two subgroups. Power calculations indicate that we could detect treatment effects of at least 1 and 0.9 percentage point for these two subsamples, respectively.

Since there are siblings in the data, we will remove from the main analysis cases when not all siblings are assigned to the same treatment conditions. Depending on how many siblings there are, we also plan to estimate within-family's externalities of the nudges, taking advantage of that sub-sample.

Empirical analysis

Since the intervention is randomly assigned, comparing treatment and control groups yields treatment effects of the SMS nudges on the outcomes of interest (Section III). Using ordinary least squares regressions, we will estimate: $Y_s^j mi = \beta_0 + \beta_1 T_s m +$

$\theta_s + \mu_s mi$ Where:

- $Y_s^j mi$: Outcome variable j for student i at school m and stratum s;
- T_m: Indicator variable equal to 1 if students I in school m and stratum s is assigned to receive SMS nudges, 0 otherwise;
- θ_s: stratum fixed effects.

We cluster standard errors at the school level, since that is the level at which the intervention is randomly assigned. We are interested in testing $\beta_1 = 0$.

B Supplementary Figures and Tables

B.1 Descriptive Statistics

This Appendix presents descriptive statistics. Table 4 describes pairwise correlations between our summary measure of standardised test scores (Panel a; also broken down by Portuguese in Panel b and math in Panel c) and whether schools were below the median of report card math and Portuguese grades by Q1/2020, and whether they offered online activities in 2019 (according to the Brazilian school census). Next, Figures 2 and 3 plot the distribution of actual and standardized Portuguese and Math test scores. Last, Table 5 compiles intraclass correlations for different outcome variables, relevant for the computation of statistical power discussed in the main text.

Supplementary Table 4
Correlation table

**Panel (a): Summary std. test scores**	
Low Q1 math	-0.211
	(0.000)
Low Q1 Portuguese	-0.360
	(0.000)
Online activities	-0.003
	(0.947)
**Panel (b): Portuguese std. test scores**	
Low Q1 math	-0.186
	(0.000)
Low Q1 Portuguese	-0.306
	(0.000)
Online activities	-0.004
	(0.930)
**Panel (c): Math std. test scores**	
Low Q1 math	-0.174
	(0.000)
Low Q1 Portuguese	-0.310
	(0.000)
Online activities	0.005
	(0.919)

Note: Pairwise correlations between standardised test scores and low (below median) baseline grades in math and Portuguese as well as the presence of online activities pre-pandemic for the control group. Panel (a) considers our summary measure (arithmetic mean), while panels (b) and (c) consider Portuguese and math test scores respectively (standardised).

Supplementary Figure 2
Distribution of Scores in the Standardized Assessment

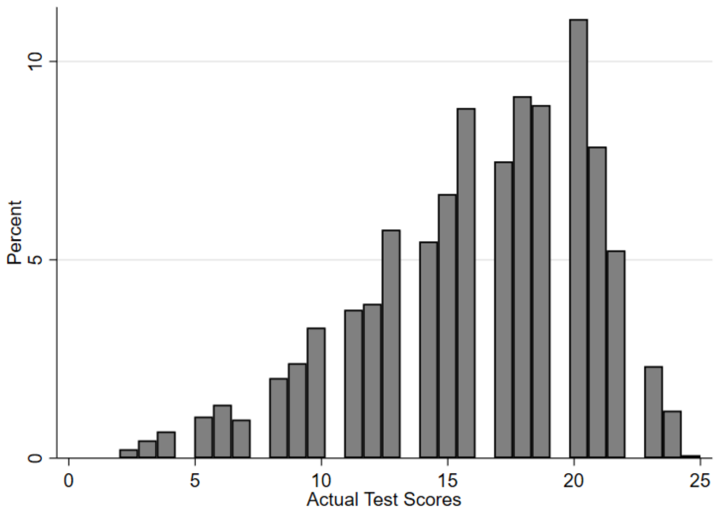

(a) *Portuguese*

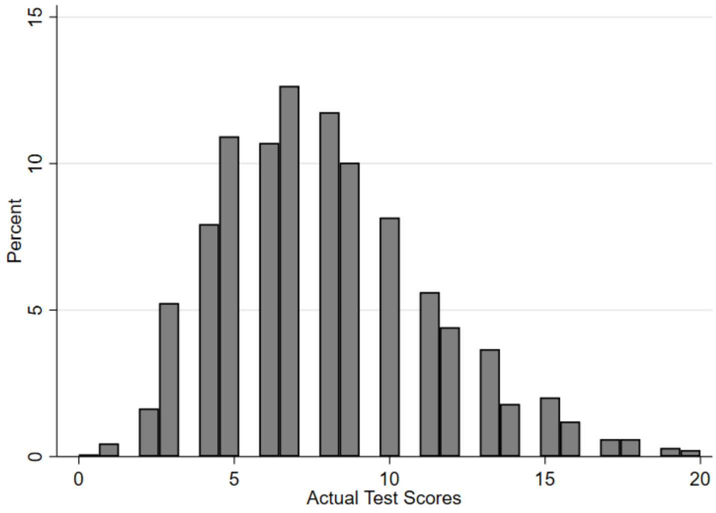

(b) *Math*

Notes: Panels (a) and (b) present the distribution of test scores obtained by high-school seniors who participated in the standardised assessment.

Supplementary Figure 3
Distribution of Standardised Test Scores

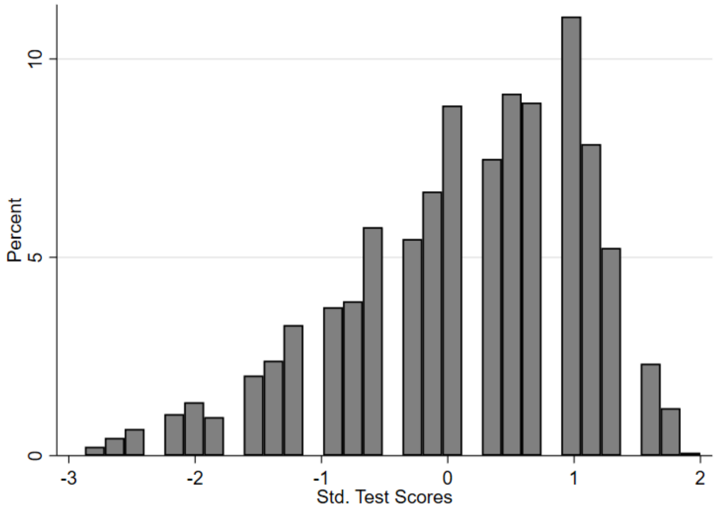

(a) *Portuguese*

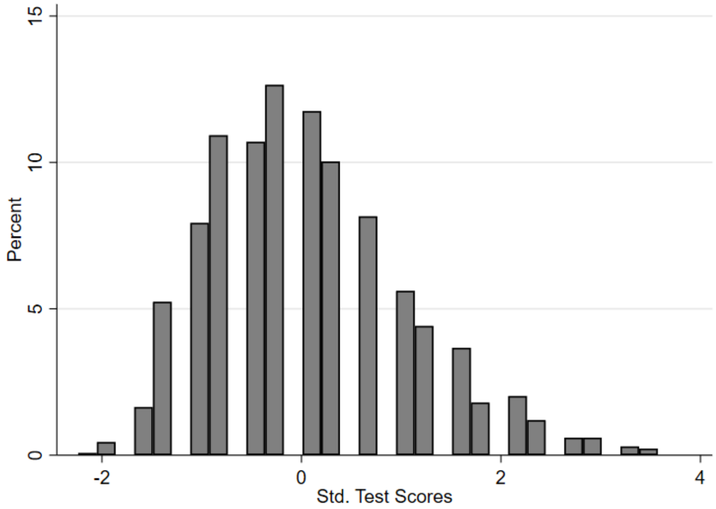

(b) *Math*

Notes: Panels (a) and (b) presents the distribution of (standardised) test scores obtained by high-school seniors in the standardised assessment. Test scores are standardised relative to the control group mean, such that the control group mean is 0 with standard deviation 1.

Supplementary Table 5
Intra-class Correlations

	ICC
	(1)
Dropout risk	0.019
Standardised scores, Portuguese	0.117
Standardised scores, math	0.095
Standardised scores, summary	0.131
Statewide scores, Portuguese	0.352
Statewide scores, math	0.421
Proficiency, Portuguese	0.185
Proficiency, Math	0.126

Note: ICCs from analysis of variance (ANOVA) for our outcome variables. Dropout = 1 for students missing Q4 report card grades in both math and Portuguese. We consider standardised scores from in math and Portuguese, as well as their summary measure (the arithmetic mean of the two). These scores are standardised relative to control group and grade to have a mean 0 and standard deviation 1. We also consider scores from State-wide exams in math and Portuguese as well as the sub-proficient school indicator (= 1 if scores < 250, and 0 otherwise).

B.2 Balance and Selective Attrition Tests

This Section compiles balance tests and selective attrition tests, showing that student and schools characteristics are balanced across the treatment and control groups and across the different arms of the content experiments, and that attrition is not systematically affected by the intervention or by content variations.

Supplementary Table 6
Balance tests: full sample

	Control	Treatment	T-test p-value	Observations
	(1)	(2)	(3)	(4)
**Panel A: Students**				
Female	0.524	0.539	0.176	18028
10th grader	0.413	0.428	0.427	18028
11th grader	0.332	0.318	0.214	18028
12th grader	0.255	0.255	1.000	18028
Phone ownership	0.408	0.422	0.702	18028
Test Score Summary Measure Q1/2020	-0.001	-0.044	0.606	17958
F-test [p-value]			0.356	17958
**Panel B: Schools**				
Online learning	0.733	0.842	0.256	87
School devices with internet access for students	0.483	0.509	0.822	86
Internet access for student use	0.500	0.632	0.246	87
Access to drinking water	1.000	0.930	0.043	87
Sanitary sewage - Public network	0.733	0.789	0.569	87
Bathroom	1.000	0.982	0.321	87
Library	1.000	0.965	0.158	87
Science lab	0.633	0.491	0.206	87
Computer Lab	0.867	0.807	0.469	87
Sports Court	0.933	0.877	0.381	87
Portuguese score in 2018 (12th grade)	279.8	282.1	0.606	55
Portuguese score in 2019 (12th grade)	286.1	283.9	0.587	57
Math score in 2018 (12th grade)	283.6	284.2	0.921	55
Math score in 2019 (12th grade)	286.2	287.5	0.776	57
F-test [p-value]			0.919	53

Notes: Panel A gives statistics for student variables. Panel B is based on 2019 Brazilian School Census and SAEGO dataset at the school level. P-values in Panel A were computed with standard errors clustered at the school level.

Supplementary Table 7
Balance tests: sub-sample with valid standardized test scores

	Control	Treatment	T-test p-value	Observations
	(1)	(2)	(3)	(4)
**Panel A: Students**				
Female	0.519	0.547	0.369	1336
11th grader	0.989	0.989	0.959	1336
12th grader	0.011	0.011	0.959	1336
Phone ownership	0.353	0.391	0.460	1336
Test Score Summary Measure Q1/2020	-0.000	0.077	0.575	1336
F-test [p-value]			0.770	1336
**Panel B: Schools**				
Online learning	0.731	0.851	0.245	73
School devices with internet access for students	0.520	0.574	0.664	72
Internet access for student use	0.538	0.702	0.177	73
Access to drinking water	1.000	0.936	0.082	73
Sanitary sewage - Public network	0.731	0.830	0.345	73
Bathroom	1.000	0.979	0.322	73
Library	1.000	0.979	0.322	73
Science lab	0.692	0.553	0.241	73
Computer Lab	0.923	0.787	0.096	73
Sports Court	0.923	0.872	0.486	73
Portuguese score in 2018 (12th grade)	281.5	282.1	0.885	54
Portuguese score in 2019 (12th grade)	286.1	283.9	0.587	57
Math score in 2018 (12th grade)	285.0	284.2	0.881	54
Math score in 2019 (12th grade)	286.2	287.5	0.776	57
F-test [p-value]			0.919	53

Notes: Students and schools considered are only the ones that had students that made the exam in Q2/2021. Panel A gives statistics for student variables. Panel B is based on 2019 Brazilian School Census and SAEGO dataset at the school level. P-values in Panel A were computed with standard errors clustered at the school level.

Supplementary Table 8

Balance tests across treatment arms of the content experiments: full sample

	Framing Gain & Social Pressure (1)	Framing Gain & No Social Pressure (2)	Framing Loss & Social Pressure (3)	Framing Loss & No Social Pressure (4)	ANOVA test p-value	Observations
Female	0.534	0.538	0.545	0.541	0.682	11897
Phone ownership	0.418	0.416	0.433	0.422	0.192	11897
Test Score Summary Measure Q1/2020	-0.020	-0.042	-0.070	-0.044	0.099	11829
F-test [p-value]					0.139	11829

Notes: Social Pressure is the treated arm for students that received the social pressure message stating that 80% of their fellow students wanted to return to in-person classes after school reopening. No Social Pressure is the treatment arm for students that received a message that just stated the importance of returning to in-person classes without reference to or data on peers' motivation to do so. Framing Loss is the treatment for messages framing the motivation to stay in school in terms of losses. Framing gain is the equivalent with motivation in terms of gains. P-values were computed considering classroom fixed effects and standard errors clustered at the school level.

Supplementary Table 9

Balance tests across treatment arms of the content experiments: sub-sample with valid standardized test scores

	Framing Gain & Social Pressure (1)	Framing Gain & No Social Pressure (2)	Framing Loss & Social Pressure (3)	Framing Loss & No Social Pressure (4)	ANOVA test p-value	Observations
Female	0.576	0.527	0.593	0.494	0.081	883
Phone ownership	0.416	0.393	0.375	0.379	0.879	883
Test Score Summary Measure Q1/2020	0.112	0.042	0.126	0.127	0.893	883
F-test [p-value]					0.267	883

Notes: Only students that had made the exam in Q2/2021 were considered. Social Pressure is the treated arm for students that received the social pressure message stating that 80% of their fellow students wanted to return to in-person classes after school reopening. No Social Pressure is the treatment arm for students that received a message that just stated the importance of returning to in-person classes without reference to or data on peers' motivation to do so. Framing Loss is the treatment for messages framing the motivation to stay in school in terms of losses. Framing gain is the equivalent with motivation in terms of gains. P-values were computed considering classroom fixed effects and standard errors clustered at the school level.

Supplementary Table 10
Selective attrition tests

	Missing Portuguese		Missing Math	
	(1)	(2)	(3)	(4)
Nudges	-0.000	0.001	-0.000	0.001
	(0.010)	(0.002)	(0.010)	(0.002)
Control Mean	0.926	0.926	0.926	0.926
R-squared	0.000	0.058	0.000	0.058
IPW	No	Yes	No	Yes
Baseline Controls	No	Yes	No	Yes
Observations	18069	17716	18069	17716

Notes: Linear probability model for missing dummies. Missing Portuguese is a dummy variable equal to one for students that did not have the 2021 test score in Portuguese. Missing Math is the equivalent of the 2021 math test score. Nudges = 1 in schools where students were nudged, and 0 otherwise. Propensity scores are predicted through a logit regression of a dummy equal to one if the student made the Q2/2021 test on student baseline covariates: gender, grade, telephone access, test score summary measure in Q1/2020, and baseline school covariates: dummy for the presence of online activities, devices with internet access for students, internet access for student use, access to drinking water, sanitary sewage - public network, bathroom, library, science lab, computer lab, and sports court. Baseline controls are the same covariates used for the Propensity Scores estimation. Standard errors are clustered at the school level.
*** p<0.01, ** p<0.05, * p<0.10

Supplementary Table 11

Selective attrition tests across treatment arms of the content experiment

	Missing Portuguese		Missing Math	
	(1)	(2)	(3)	(4)
No Social Pressure	0.010*	0.002	0.010*	0.002
	(0.006)	(0.003)	(0.006)	(0.003)
Framing Loss	0.005	-0.002	0.005	-0.002
	(0.006)	(0.003)	(0.006)	(0.003)
No Social Pressure · Framing Loss	-0.016**	0.001	-0.016**	0.001
	(0.008)	(0.004)	(0.008)	(0.004)
Control Mean	0.926	0.926	0.926	0.926
F-test No Social Pressure=Framing Loss=Interaction=0 [p-value]	0.141	0.608	0.141	0.608
R-squared	0.000	0.062	0.000	0.062
IPW	No	Yes	No	Yes
Baseline Controls	No	Yes	No	Yes
Observations	11927	11859	11927	11859

Notes: Linear probability model for missing dummies. Missing Portuguese is a dummy variable equal to one for students that did not have the 2021 test score in Portuguese. Missing Math is the equivalent of the 2021 math test score. No Social Pressure is a dummy equal to one for treated students that received message that just stated the importance of returning to in-person classes without reference to or data on peers' motivation to do so. Framing Loss is a dummy for treated students that received the message framing the motivation to stay in school in terms of losses. Experiment controls were dropped in the regression. Propensity scores are predicted through a logit regression of a dummy equal to one if the student made the Q2/2021 test on student baseline covariates: gender, grade, telephone access, test score summary measure in Q1/2020, and baseline school presence of: online activities, devices with internet access for students, internet access for student use, access to drinking water, sanitary sewage - public network, bathroom, library, science lab, computer lab, and sports court. Baseline controls are the same covariates used for the Propensity Scores estimation. Standard errors are clustered at the school level
*** p<0.01, ** p<0.05, * p<0.10

B.3 Short-Run Effects on students' Motivation and Attendance

This Section complements the analyses in the main text by estimating treatment effects on students' motivation to engage with school activities shortly after the onset of the intervention. These additional results help us provide direct evidence that the treatment effects on learning outcomes that we document are caused by the impacts of the intervention on students' socio-emotional skills and motivation targeted by the text messages.

Figure 4 estimates average treatment effects of the intervention on an indicator variable equal to 1 if the student attended no classes in the two weeks before the winter break, and 0 otherwise, based on administrative data. Despite quality issues for quarterly attendance data, we were able to obtain daily administrative data on attendance for each student in our sample during June 2020. Such data was in fact made available for the vast majority of students (see the Supplementary Materials). Prolonged absenteeism is a well-known predictor of student dropouts. The figure showcases that while 7.21% of students in the control group had not followed remote learning activities right before the winter break, that figure was only 0.33% in the treatment group – an over 95% reduction (p-value = 0.00).

Figure 5 estimates average treatment effects on an indicator variable equal to 1 if the student states that they do not want to go back to school once in-person classes return, and 0 otherwise, using self-reported data. Panel A displays weekly averages for the treatment and control groups, and Panel B estimates week-by-week treatment effects. Panel 5a documents a striking pattern for lack of motivation to return to in-person classes in the control group, which increased more than 2-fold in little over a month (starting from 15% by the 2nd week of June and reaching 39% by the 3rd week of July). Panel 5a also shows that lack of motivation to return to in-person

classes not only started from a lower level in the treatment group already by week 2, but also increased at slower rates while the intervention lasted. 5b confirms those patterns: the intervention decreased lack of motivation to return to in-person classes by over 30% already by week 2, and effect sizes persisted into the winter break (significant at the 5% level by week 4, and significant at the 10% level in week 5).

After communication temporarily stopped during the winter break, treatment effects gradually faded out. Panel 5a in Figure 5 shows that lack of motivation to return to in-person classes increased nearly by the same rate across the treatment and control groups during the winter break. Panel 5b documents that effect sizes gradually declined, from 40% to 20% only two weeks later, and no longer statistically different from zero.

All in all, these patterns confirm that students react to the messages both when it comes to their motivation to return to in-person classes and when it comes to attendance in remote learning activities right before the winter break.

Supplementary Figure 4

Treatment effects on prolonged absenteeism right before winter break

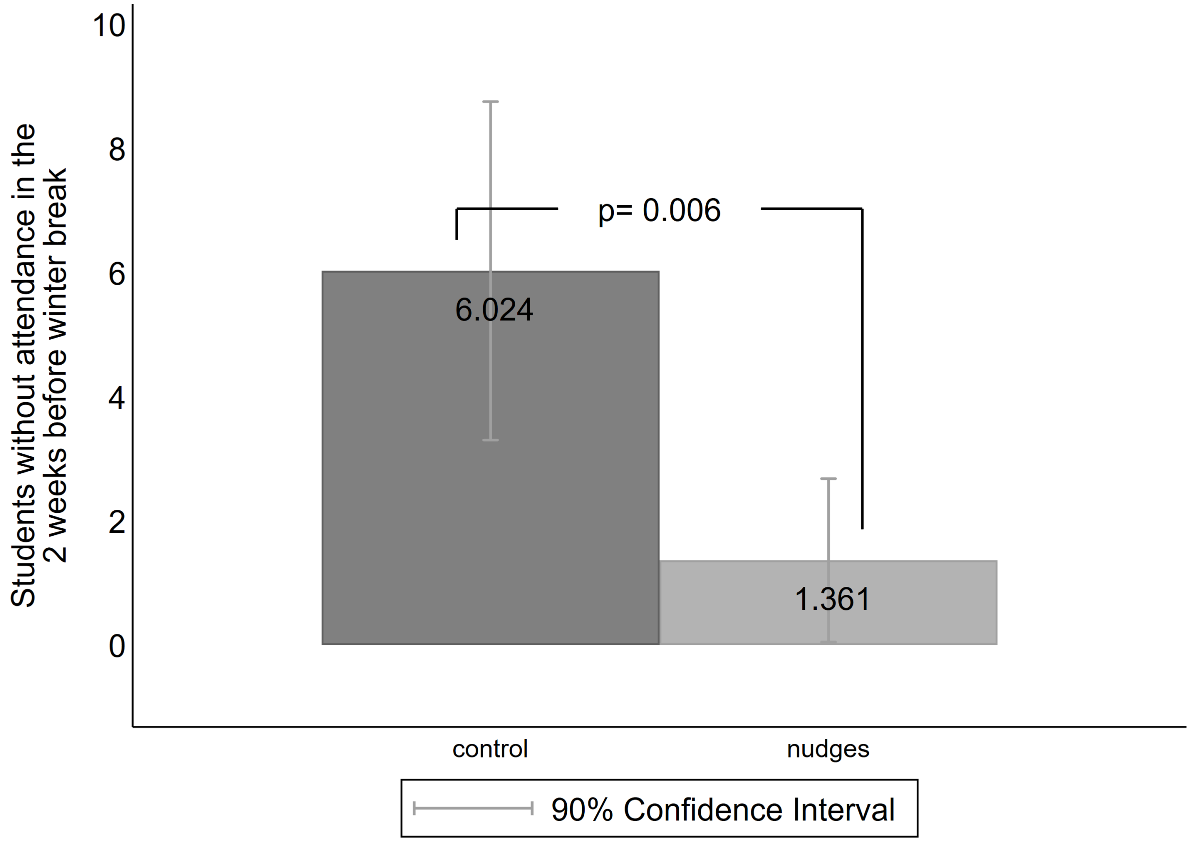

Notes: ITT estimate from an Ordinary Least Squares (OLS) regression with dependent variable = 1 if a student had no attendance on record over the last two weeks before the winter break, and 0 otherwise. Treatment = 1 in schools where students received text messages targeting their socio-emotional skills, and 0 otherwise. Propensity score re-weighting is employed to ensure that student characteristics are balanced across the treatment and control groups. Propensity scores are predicted through a logit regression of treatment on baseline characteristics (gender, grade, and telephone access). 90% confidence intervals in light grey brackets; p-value in dark grey brackets from an OLS regression with standard errors clustered at the school level.

Supplementary Figure 5
Treatment effects on students' motivation to return to school

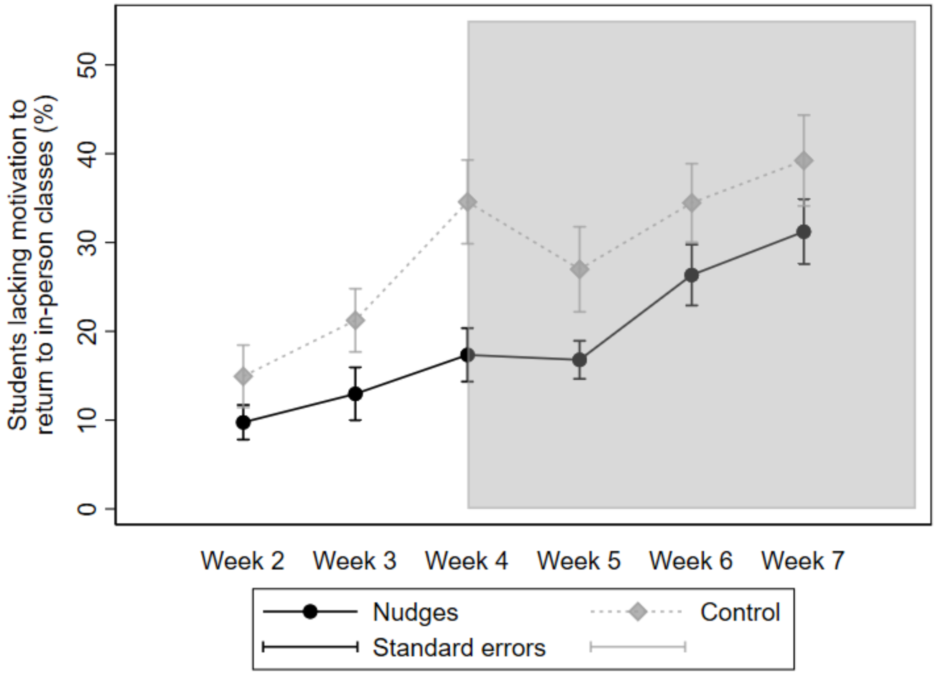

(a) *Students' lack of motivation to return to school once they reopen (based on self-reported data) for the treatment and control groups, week by week*

Notes: Weekly sample averages for lack of motivation to return to in-person classes (= 1 if the student states that they do not think they will be back in school when in-person classes resume, and 0 otherwise) for the treatment group (in black) and for the control group (in light grey). Propensity score re-weighting is employed to ensure that student characteristics are balanced across the treatment and control groups. Propensity scores are predicted through a logit regression of treatment on baseline characteristics (gender, grade, and telephone access). Self-reports based on weekly SMS surveys from rotating sub-samples of students in the treatment and control groups, from the week after the intervention started until 3 weeks after it ended. Standard errors clustered at the school level. The shaded area corresponds to the weeks during the winter break, when no messages were sent.

Supplementary Figure 5
Treatment effects on students' motivation to return to school

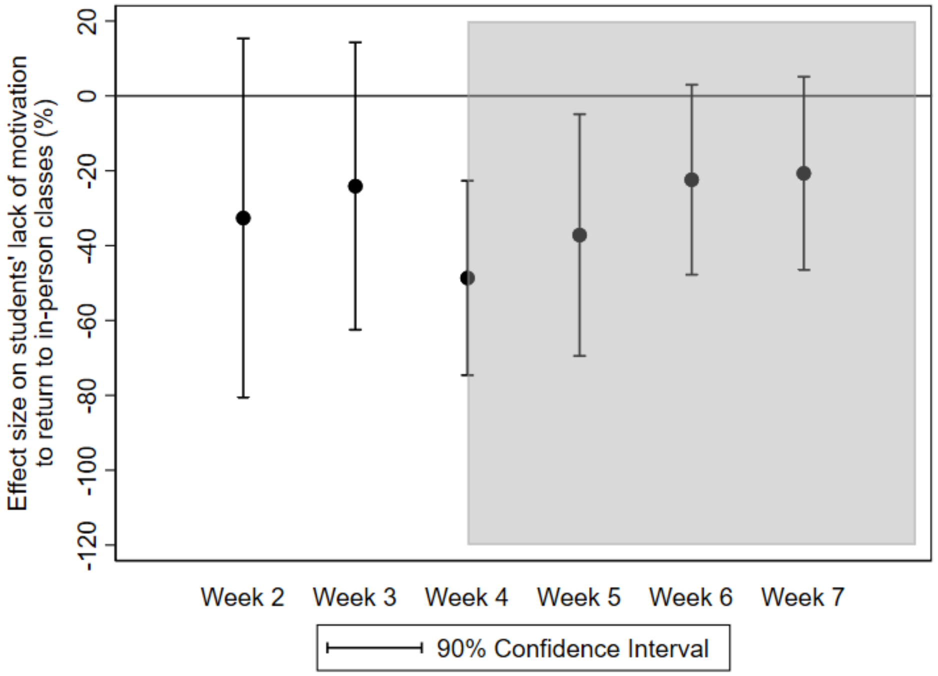

(b) *Treatment effects on students' lack of motivation to return to school once they reopen (based on self-reported data), week by week*

Notes: ITT estimates from Ordinary Least Squares (OLS) regressions for lack of motivation to return to in-person classes (= 1 if the student states that they do not think they will be back in school when in-person classes resume, and 0 otherwise) for the treatment group (in black) and for the control group (in light grey). Propensity score re-weighting is employed to ensure that student characteristics are balanced across the treatment and control groups. Propensity scores are predicted through a logit regression of treatment on baseline characteristics (gender, grade, and telephone access). Self-reports based on weekly SMS surveys from rotating sub-samples of students in the treatment and control groups, from the week after the intervention started until 3 weeks after it ended. Standard errors clustered at the school level. The shaded area corresponds to the weeks during the winter break, when no messages were sent.

C Supplementary References

Allen, J. P., Pianta, R. C., Gregory, A., Mikami, A. Y., & Lun, J. (2011). An interaction-based approach to enhancing secondary school instruction and student achievement. *Science, 333*(6045), 1034-1037.

Azmat, G., & Iriberri, N. (2010). The importance of relative performance feedback information: Evidence from a natural experiment using high school students. *Journal of Public Economics, 94*(7-8), 435-452.

Barrera-Osorio, F., Gonzalez, K., Lagos, F., & Deming, D. J. (2020). Providing performance information in education: An experimental evaluation in Colombia. *Journal of Public Economics, 186*, 104185.

Bergman, P. (2021). Parent-child information frictions and human capital investment: Evidence from a field experiment. *Journal of Political Economy, 129*(1), 286-322.

Bettinger, E., Cunha, N., Lichand, G., Madeira, R. (2020). *"Are Effects of Informational Interventions Driven by Salience?".* Working Paper.

Bordalo, P., Gennaioli, N., & Shleifer, A. (2020). Memory, Attention, and Choice. *The Quarterly Journal of Economics, 135*(3), 1399-1442.

Bryan, C. J., Yeager, D. S., Hinojosa, C. P., Chabot, A., Bergen, H., Kawamura, M., & Steubing, F. (2016). Harnessing adolescent values to motivate healthier eating. *Proceedings of the National Academy of Sciences, 113*(39), 10830-10835.

Bursztyn, L., & Jensen, R. (2015). How does peer pressure affect educational investments?. *The quarterly journal of economics, 130*(3), 1329-1367.

Castillo, M., Ferraro, P. J., Jordan, J. L., & Petrie, R. (2011). The today and tomorrow of kids: Time preferences and educational outcomes of children. *Journal of Public Economics, 95(11-12), 1377-1385.*

Castleman, B. L., & Page, L. C. (2015). Summer nudging: Can personalized text messages and peer mentor outreach increase college going among low-income high school graduates? *Journal of Economic Behavior Organization, 115, 144–160.*

DellaVigna, S., Linos, E. (2020). *RCTs to Scale: Comprehensive Evidence from Two Nudge Units.* Working Paper, UC Berkeley.

Dizon-Ross, R. (2019). Parents' beliefs about their children's academic ability: Implications for educational investments. *American Economic Review, 109*(8), 2728-65.

Gennetian, L. A., & Shafir, E. (2015). The persistence of poverty in the context of financial instability: A behavioral perspective. textitJournal of Policy Analysis and Management, 34(4), 904-936.

Goodenow, C., & Grady, K. E. (1993). The relationship of school belonging and friends' values to academic motivation among urban adolescent students. *The Journal of Experimental Education, 62*(1), 60-71.

Hanushek, E. A., & Woessmann, L. (2008). The role of cognitive skills in economic development. *Journal of economic literature, 46*(3), 607-68.

Jalava, N., Joensen, J. S., & Pellas, E. (2015). Grades and rank: Impacts of non-financial incentives on test performance. *Journal of Economic Behavior & Organization, 115*, 161-196.

Karlan, D., McConnell, M., Mullainathan, S., & Zinman, J. (2016). Getting to the top of mind: How reminders increase saving. *Management Science, 62*(12), 3393-3411.

Kiefer, S. M., Alley, K. M., & Ellerbrock, C. R. (2015). Teacher and peer support for young adolescents' motivation, engagement, and school belonging. *Rmle Online, 38*(8), 1-18.

Lichand, G., Wolf, S. (2020). *"Are Parenting Interventions Transferable Across Settings? Evaluating Key Constraints in Sub-Saharan Africa".* Working Paper.

Mani, A., Mullainathan, S., Shafir, E., & Zhao, J. (2013). Poverty impedes cognitive function. *science, 341(6149), 976-980.*

Mullainathan, S., & Shafir, E. (2013). Scarcity: Why having too little means so much. *Times Books/Henry Holt and Co.*

Riener, G., & Wagner, V. (2019). On the design of non-monetary incentives in schools. *Education Economics, 27*(3), 223-240.

Rogers, F. H., Sabarwal, S. (2020). *The COVID-19 Pandemic: Shocks to Education and Policy Responses* (No. 148198, pp. 1-0). The World Bank.

SARESP (2014a). *Relatório Pedagógico. Língua Portuguesa.*

SARESP (2014b). *Relatório Pedagógico. Matemática.*

Siebert, Stanley, Ho Lun Wong, Xiangdong Wei, & Xiang Zhou. (2018). Student feedback, parent-teacher communication, and academic performance: Experimental evidence from rural China. *IZA working paper No. 11347.*

Sutter, M., Kocher, M. G., Glätzle-Rützler, D., & Trautmann, S. T. (2013). Impatience and

uncertainty: Experimental decisions predict adolescents' field behavior. *American Economic Review, 103(1), 510-31.*

Vegas, E. (2020). School closures, government responses, and learning inequality around the world during COVID-19. *Washington, DC: Brookings Institution.*

Wentzel, K. R. (1998). Social relationships and motivation in middle school: The role of parents, teachers, and peers. *Journal of educational psychology, 90*(2), 202.

Yeager, D. S., Walton, G. M., Brady, S. T., Akcinar, E. N., Paunesku, D., Keane, L., ... & Dweck, C. S. (2016). Teaching a lay theory before college narrows achievement gaps at scale. *Proceedings of the National Academy of Sciences, 113*(24), E3341-E3348.
