# Supplementary material for: Neglecting students’ socio-emotional skills magnified learning losses during the pandemic
Source: NPJ Sci Learn. 2024 Apr 9;9:28. doi: 10.1038/s41539-024-00235-9 (PMC11003960; doi:10.1038/s41539-024-00235-9)
Supplement: Supplementary file 2 — Reporting summary [file 41539_2024_235_MOESM2_ESM.pdf]

nature portfolio

Corresponding author(s): Guilherme Lichand

Last updated by author(s): Mar 1, 2024

Reporting Summary

Nature Portfolio wishes to improve the reproducibility of the work that we publish. This form provides structure for consistency and transparency in reporting. For further information on Nature Portfolio policies, see our Editorial Policies and the Editorial Policy Checklist.

Statistics

For all statistical analyses, confirm that the following items are present in the figure legend, table legend, main text, or Methods section.

n/a	Confirmed	
☐	☒	The exact sample size (*n*) for each experimental group/condition, given as a discrete number and unit of measurement
☐	☒	A statement on whether measurements were taken from distinct samples or whether the same sample was measured repeatedly
☐	☒	The statistical test(s) used AND whether they are one- or two-sided<br>*Only common tests should be described solely by name; describe more complex techniques in the Methods section.*
☐	☒	A description of all covariates tested
☐	☒	A description of any assumptions or corrections, such as tests of normality and adjustment for multiple comparisons
☐	☒	A full description of the statistical parameters including central tendency (e.g. means) or other basic estimates (e.g. regression coefficient) AND variation (e.g. standard deviation) or associated estimates of uncertainty (e.g. confidence intervals)
☐	☒	For null hypothesis testing, the test statistic (e.g. *F*, *t*, *r*) with confidence intervals, effect sizes, degrees of freedom and *P* value noted<br>*Give P values as exact values whenever suitable.*
☒	☐	For Bayesian analysis, information on the choice of priors and Markov chain Monte Carlo settings
☐	☒	For hierarchical and complex designs, identification of the appropriate level for tests and full reporting of outcomes
☐	☒	Estimates of effect sizes (e.g. Cohen's *d*, Pearson's *r*), indicating how they were calculated

Our web collection on statistics for biologists contains articles on many of the points above.

Software and code

Policy information about availability of computer code

Data collection	We did not collect data directly for this study. Access to all data on students' attendance, report card grades and standardized test scores (all without PIIs) was granted by the Goiás Secretariat of Education.
Data analysis	We analyzed all data using Stata.

For manuscripts utilizing custom algorithms or software that are central to the research but not yet described in published literature, software must be made available to editors and reviewers. We strongly encourage code deposition in a community repository (e.g. GitHub). See the Nature Portfolio guidelines for submitting code & software for further information.

Data

Policy information about availability of data

All manuscripts must include a data availability statement. This statement should provide the following information, where applicable:

- Accession codes, unique identifiers, or web links for publicly available datasets
- A description of any restrictions on data availability
- For clinical datasets or third party data, please ensure that the statement adheres to our policy

The data that support the findings of this study (without PIIs), along with complete replication package, are available at https://osf.io/3sqfr/.

Research involving human participants, their data, or biological material

Policy information about studies with human participants or human data. See also policy information about sex, gender (identity/presentation), and sexual orientation and race, ethnicity and racism.

Reporting on sex and gender	In Table 3, column 3 documents that boys and girls did not systematically endure differential learning losses in control schools. Similarly, the effect of the intervention did not significantly vary by student gender.
Reporting on race, ethnicity, or other socially relevant groupings	We had no access to student race or ethnicity information.
Population characteristics	We only had access to student gender, grade and phone ownership information, besides data on their attendance, report card grades, and standardized test scores.
Recruitment	We did not recruit participants for this research. Rather, access to all data on students' attendance, report card grades and standardized test scores (all without PIIs) was granted by the Goiás Secretariat of Education.
Ethics oversight	Approval for this study was obtained from the Institutional Review Board of the Department of Economics at the University of Zurich (2020-033). When it comes to informed consent, since participants are minors, broad consent was obtained from their legal guardians directly by the Education Secretariat (at the time of school enrollment), allowing researchers to use secondary information from administrative records without eliciting further consent. Our implementing partner further obtained students' assent directly via text messages (SMS): participants were informed and reminded of the fact that they could opt-out from the intervention and SMS surveys at any point (by simply replying 'STOP' or 'CANCEL', free of charge), without consequence.

Note that full information on the approval of the study protocol must also be provided in the manuscript.

Field-specific reporting

Please select the one below that is the best fit for your research. If you are not sure, read the appropriate sections before making your selection.

☐ Life sciences ☒ Behavioural & social sciences ☐ Ecological, evolutionary & environmental sciences

For a reference copy of the document with all sections, see nature.com/documents/nr-reporting-summary-flat.pdf

Behavioural & social sciences study design

All studies must disclose on these points even when the disclosure is negative.

Study description	Quantitative study.
Research sample	Full-time high-school students in the State of Goiás, Brazil.
Sampling strategy	The Education Secretariat had access to valid phone numbers for 18,256 students, roughly 40% of the total. We do not have data on students outside our sample.
Data collection	We did not recruit participants for this research. Rather, access to all data on students' attendance, report card grades and standardized test scores (all without PIIs) was granted by the Goiás Secretariat of Education.
Timing	The intervention started on June 9, 2020 (during Q2/2020), and continued until July 7, 2021 (at the end of Q2/2021). No messages were sent during the winter break in July, 2020. Standardized tests (in-person) were conducted in Q2/2021 (between April and May) with a random sample of full-time high-school seniors in the State.
Data exclusions	We have 2021 data on school-level Portuguese and math proficiency levels for high-school seniors in 58 out of the 87 schools that are part of our experiment. The reason for missing data is that the State does not disclose school-level proficiency levels when less than 80% of the students in a school take the exam. 1 of these schools had no data for 2019, and 3 of them, no data for 2018. As such, we have 170 observations for each subject for the 2018-2021 period, and 340 when we pool math and Portuguese proficiency levels in our analyses. Out of these 58 high schools, 38 also had data for 9th-graders' proficiency levels in 2021 (27 in 2019 and 29 in 2018), which we use to estimate placebo effects. Pooling math and Portuguese proficiency levels, we have 188 observations in that case. Last, when we pool data for high-school seniors and 9th-graders in the analyses of treatment and placebo effects on the share of sub-proficient schools across both subjects, we have 528 observations in total.
Non-participation	1.14% of participants opted out over the course of the study.
Randomization	In total, 12,056 high-school students across 57 public schools were randomly assigned to receive SMS nudges, while 6,200 high-school students across 30 public schools were assigned to the control group -- i.e., they did not receive nudges or other text messages from their schools over that period. Randomization was undertaken at the school level, stratified by gender, grade and phone ownership. More students were assigned to treatment than control because we split treated students into additional

treatment arms, varying the content of nudges across them. We cross-randomized treated students to (1) a framing experiment, and (2) a social pressure experiment. Randomization was undertaken at the individual level. These additional experiments took place in the first month of the intervention, during Q2/2020. All treated students received exactly the same content from Q3/2020 until the end of the intervention.

Reporting for specific materials, systems and methods

We require information from authors about some types of materials, experimental systems and methods used in many studies. Here, indicate whether each material, system or method listed is relevant to your study. If you are not sure if a list item applies to your research, read the appropriate section before selecting a response.

Materials & experimental systems

n/a	Involved in the study
☒	☐ Antibodies
☒	☐ Eukaryotic cell lines
☒	☐ Palaeontology and archaeology
☒	☐ Animals and other organisms
☒	☐ Clinical data
☒	☐ Dual use research of concern
☒	☐ Plants

Methods

n/a	Involved in the study
☒	☐ ChIP-seq
☒	☐ Flow cytometry
☒	☐ MRI-based neuroimaging

Plants

Seed stocks

Report on the source of all seed stocks or other plant material used. If applicable, state the seed stock centre and catalogue number. If plant specimens were collected from the field, describe the collection location, date and sampling procedures.

Novel plant genotypes

Describe the methods by which all novel plant genotypes were produced. This includes those generated by transgenic approaches, gene editing, chemical/radiation-based mutagenesis and hybridization. For transgenic lines, describe the transformation method, the number of independent lines analyzed and the generation upon which experiments were performed. For gene-edited lines, describe the editor used, the endogenous sequence targeted for editing, the targeting guide RNA sequence (if applicable) and how the editor was applied.

Authentication

Describe any authentication procedures for each seed stock used or novel genotype generated. Describe any experiments used to assess the effect of a mutation and, where applicable, how potential secondary effects (e.g. second site T-DNA insertions, mosiacism, off-target gene editing) were examined.
